# Design principles for water dissociation catalysts in high-performance bipolar membranes

**DOI:** 10.1038/s41467-022-31429-7

**Published:** 2022-07-04

**Authors:** Lihaokun Chen, Qiucheng Xu, Sebastian Z. Oener, Kevin Fabrizio, Shannon W. Boettcher

**Affiliations:** 1 Department of Chemistry and Biochemistry and the Oregon Center for Electrochemistry, University of Oregon, Eugene, OR 97403 USA; 2 Present Address: Surface Physics and Catalysis (Surf Cat) Section, Department of Physics, Technical University of Denmark, 2800 Kgs Lyngby, Denmark; 3 Present Address: Department of Interface Science, Fritz Haber Institute of the Max Planck Society, 14195 Berlin, Germany

**Keywords:** Energy science and technology, Electrocatalysis, Electrochemistry

## Abstract

Water dissociation (WD, H_2_O → H^+^ + OH^−^) is the core process in bipolar membranes (BPMs) that limits energy efficiency. Both electric-field and catalytic effects have been invoked to describe WD, but the interplay of the two and the underlying design principles for WD catalysts remain unclear. Using precise layers of metal-oxide nanoparticles, membrane-electrolyzer platforms, materials characterization, and impedance analysis, we illustrate the role of electronic conductivity in modulating the performance of WD catalysts in the BPM junction through screening and focusing the interfacial electric field and thus electrochemical potential gradients. In contrast, the ionic conductivity of the same layer is not a significant factor in limiting performance. BPM water electrolyzers, optimized via these findings, use ~30-nm-diameter anatase TiO_2_ as an earth-abundant WD catalyst, and generate O_2_ and H_2_ at 500 mA cm^−2^ with a record-low total cell voltage below 2 V. These advanced BPMs might accelerate deployment of new electrodialysis, carbon-capture, and carbon-utilization technology.

## Abstract (web summary)

It is important yet challenging to elucidate the mechanism of water dissociation in bipolar membrane electrolysers. Here the authors show how water dissociation is accelerated by electric-field-focusing and catalytic effects and uncover design principles to optimize the performance.

## Introduction

**Fig. 1:**
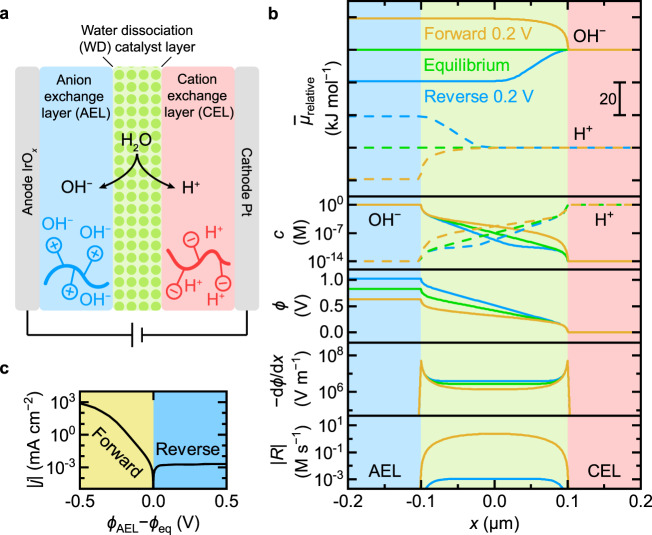
Properties of BPMs. **a** Schematic of a BPM electrolyzer. Pure water is fed through the anode and cathode gas-diffusion layers (GDLs) and diffuses into the AEL|CEL junction where water is dissociated with the aid of WD catalysts. **b** Steady-state numerical simulation results of a BPM at equilibrium (green), in forward bias 0.2 V (orange), and in reverse bias 0.2 V (blue). From top to bottom are the profiles of relative electrochemical potential ${\bar{\mu }}_{{{{{{\rm{relative}}}}}}}$, molar concentration $c$, electric potential $\phi$, electric field $-{{{{{\rm{d}}}}}}\phi /{{{{{\rm{d}}}}}}x$, and magnitude of the net reaction rate $\left|R\right|$ (sum of dissociation and recombination). At equilibrium, the electrochemical potentials of each mobile species are the same across the whole BPM. **c** Simulated polarization curve of a BPM in forward bias and reverse bias. See Methods for more information.

A bipolar membrane (BPM) consists of an anion-exchange layer (AEL) and a cation-exchange layer (CEL) sandwiched together^1,2^. The AEL contains fixed positively charged groups and mobile anions, while CEL contains fixed negatively charged groups and mobile cations (Fig. 1a). BPMs were first conceived as an ionic counterpart to current-rectifying semiconductor *pn* junctions^3–5^, and now are used in electrodialysis devices for desalination and acid/base production from brine^6^, fuel cells^7^, water and CO_2_ electrolysis^8,9^, flow batteries^10^, and protonic diodes^11^. They provide distinct alkaline and acidic environments that are ideal for water oxidation and water reduction, respectively, offering new pathways for increasing electrolyzer performance while reducing or eliminating precious metals use^12^. In CO_2_ electroreduction, BPMs retard crossover of carbonate and product species, thus increasing Faradaic efficiency^13–16^.

Water dissociation (WD, H_2_O → H^+^ + OH^−^) in reverse bias, and H^+^/OH^−^ recombination in forward bias, are key to BPM function. Despite recent advances^8,17–22^, the mechanism(s) of WD in BPMs, and many of the factors critical for controlling the WD kinetics, remain unclear. Kunst and Lovreček appear to be the first to explain high rates of WD (relative to bulk water) via electric-field enhancement at the AEL|CEL interface via the second Wien effect^23^, but conclude that the field strength is too small to explain the high observed currents^24^. Simons then proposed a proton-transfer WD-catalysis mechanism at tertiary amino groups on the AEL at the BPM junction^25,26^. Strathmann et al. argued that only with a relative permittivity of water <10 at the AEL|CEL interface can the enhanced WD rate be explained by the second Wien effect, and that the ideal p*K*_a_ for WD catalysis via proton transfer is ~7, assuming a single type of acid/base group in the AEL|CEL junction^27^. Abdu et al. controlled the BPM junction WD activity and ionic selectivity with layer-by-layer deposited polyelectrolytes^18^. Recently Yan et al. fabricated custom BPMs with different amounts of graphene oxide (GO) between the AEL and CEL as WD catalyst and proposed that the catalytic and electric-field-enhancement effects play counterbalanced roles in speeding WD^19^. The best performance they found was with the largest amount of GO in the range tested (4 layers, probably ~5 nm^28^). Oener et al. discovered a link between WD in electrocatalysis and BPMs and showed how the point of zero charge (PZC) of oxide nanoparticles correlates with the (seemingly pH-dependent) WD activity^8^, while also demonstrating dramatically improved BPM performance with metal-oxide nanoparticle bilayer films ~500 nm in thickness. However, the mechanistic details of WD remain unclear, particularly the exact role of the electric field in the junction for WD catalysts with varying electronic and dielectric properties as well as thicknesses (see Supplementary Discussion). This knowledge gap slows the design of higher-performance BPMs for key energy applications.

Here we uncover new BPM design principles derived from numerical simulations and measurements on well-defined custom BPM architectures with controlled WD catalyst loading, particle size, composition, and electrical properties. We find that for semiconducting nanoparticles such as TiO_2_, there is a clear optimal range of loading/thickness, ~10–30 μg cm^−2^ (~200–600 nm in thickness), and particle size (20–30 nm), out of which the performance becomes substantially worse. For electronic conductors, including antimony-doped tin oxide (ATO), IrO_*x*_, and Pt nanoparticles, the optimal loading is much higher (>100 μg cm^−2^), and the optimal performance window is wider. Mobile electrons of the WD catalyst layer inside the (electrically disconnected) BPM junction appear to screen and focus the electric field to a narrow region at the AEL|WD-catalyst and WD-catalyst|CEL interface, speeding catalytic WD. Contrary to expectations, we find that TiO_2_ with particle sizes <20 nm decrease performance, despite providing a higher surface area over which the WD reaction can occur, which is also explained by electric-field effects. By collectively controlling these parameters, we demonstrate anatase TiO_2_ WD catalyst layers that enable BPM pure-water electrolyzers which run at 500 mA cm^−2^ at record-low voltages below 2 V.

## Results

### Simulations to inform design principles

We built a 1D numerical model with the minimum components to represent BPM features to illustrate design principles and help interpret experimental data. Taking H^+^ and OH^−^ as the only mobile ions during pure-water operation (consistent with our experiments), we simulated profiles of relative electrochemical potential ${\bar{\mu }}_{{{{{{\rm{relative}}}}}}}$, molar concentration $c$, electric potential $\phi$, electric field $-{{{{{\rm{d}}}}}}\phi /{{{{{\rm{d}}}}}}x$, and net reaction rate $R$ (Fig. 1b). Changes in the AEL and CEL are small, so we focus on the junction region. The concentration of OH^−^ drops nearly exponentially from the AEL into the junction (notice the semi-log scale), and H^+^ shows the same behavior at the CEL. This suggests that the ionic conductivity in the junction is small due to the low concentration of mobile H^+^ and OH^−^ without added ionomers. Across the junction, $\phi$ drops nearly linearly with a steeper slope (higher electric field) at the AEL|WD-catalyst and WD-catalyst|CEL interfaces due to screening by the dissociated ions from the respective ionomer layers.

The simulated polarization curve (Fig. 1c) shows rectification of ionic current. In forward bias, H^+^ and OH^−^ are driven into the junction by the gradients in electrochemical potential, leading to an increasing conductivity and their recombination to form water. In reverse bias, H^+^ and OH^−^ are driven out of the junction, decreasing the concentration and thus ionic conductivity. The polarization curve approaches a limiting current density determined by, in this simulation, the product of the water dissociation rate constant *k*_f_, the concentration of water in the junction, and the junction thickness. We note that $\phi$ drops almost entirely across the junction, since the ionic conductivity there is small compared with the AEL and CEL. Both the dissociation and recombination also occur almost exclusively in the junction region. We will return to this model in the context of the experimental data below.

### WD catalyst layer thickness/loading effects

Conventionally, BPMs are characterized in H-cells with soluble supporting electrolytes^1^. In such systems, the ionic current is due to transport of H^+^ and OH^−^ from WD and of so-called “co-ions”, i.e., electrolyte species like Na^+^ or Cl^−^. Differentiating between these two currents is difficult, and often uncontrolled pH gradients form, complicating the analysis. We use electrolyzers in a membrane-electrode-assembly (MEA) geometry fed by pure water and the current is thus carried exclusively by H^+^ and OH^−^. This MEA is under active compression so that no adhesives, interpenetrating 3D junctions, or other complicating interface structures are needed. This allows us to make fundamental discoveries as to the underlying physics and chemistry that govern the electrochemical response of BPMs and rationally design for higher performance. The total cell voltage reported includes the WD overpotential (*η*_wd_), ohmic losses, and overpotentials due to charge-transfer (CT) reactions at the electrodes, i.e., the oxygen-evolution reaction (OER) and the hydrogen-evolution reaction (HER). To compare different WD catalysts, the electrodes, gas-diffusion layers (GDLs), HER catalyst (Pt), OER catalyst (IrO_*x*_), assembly methods, temperature (55 ± 2 °C, maximum fluctuation), etc. are all kept identical (see Methods and Supplementary Fig. 1 for detailed schematics).

**Fig. 2:**
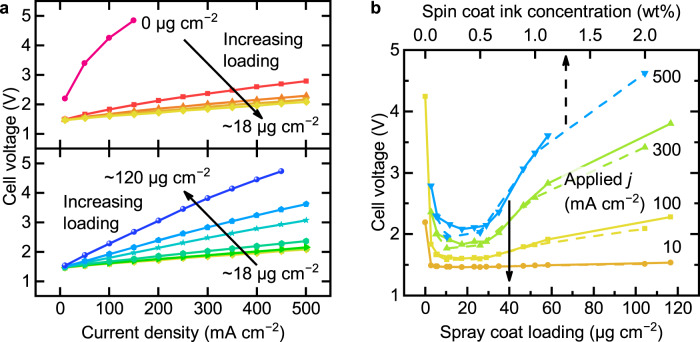
Performance of BPM electrolyzers with TiO2-P25 as WD catalysts. **a** Polarization curves of BPM electrolyzers with different loadings of TiO_2_-P25 WD catalyst deposited by spray coating. **b** Cell voltage of BPM electrolyzers as a function of spray-coating loading (solid lines) of TiO_2_-P25 and spin-coating ink concentration (dashed lines) of TiO_2_-P25 at different applied current densities. The 2.0 wt% sample is 1.0 wt% ink spun twice. The temperature is 55 ± 2 °C (maximum fluctuation).

We first studied TiO_2_-P25 as a benchmark WD catalyst as it is commercially available at low cost, has good WD performance, and is chemically stable in both acid and base^8,9^. Increasing the spray-coated loading of TiO_2_-P25 from 0 to ~18 μg cm^−2^ decreases the cell voltage, while higher loading increases the voltage (Fig. 2a,  2b, solid lines, and Supplementary Fig. 2). The polarization curves are found to be nearly linear, consistent with catalyzed WD and a low driving force needed for WD. The reproducibility of the BPM electrolyzers is verified with ~18 μg cm^−2^ TiO_2_-P25 samples at different testing dates with different batches of GDLs (Supplementary Fig. 3). The cell voltage at 500 mA cm^−2^ is 2.05 ± 0.06 V (standard deviation across 7 samples).

**Fig. 3:**
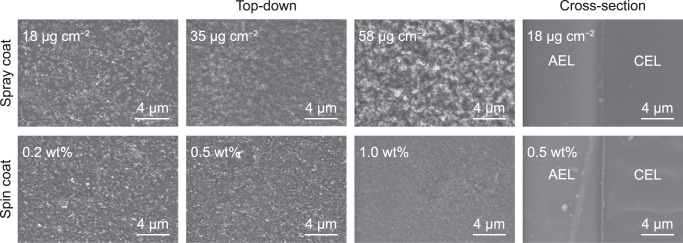
SEM images of TiO2-P25 on the CEL and BPM cross-sections. For spray-coated samples, the approximate loadings are given while for spin-coated samples, the ink concentrations are given. The optimal spin-coated WD catalysts layers are smoother, with more-uniform coverage, but only marginally improved performance.

The best-performing BPMs had incomplete coverage of spray-coated TiO_2_-P25 (Fig. 3), which led us to question whether this was important for function. We thus also spin-coated more-uniform TiO_2_-P25 films and found a comparable loading dependence (Fig. 2b, dash lines). The best performance was made from an ink with 0.2 wt% TiO_2_, and resulted in the membrane uniformly covered with TiO_2_-P25 at a thickness comparable to the spray-coated sample with ~18 μg cm^−2^ (Fig. 3). With the 0.5 wt% ink, the performance is similar, and uncovered membrane regions are not evident. Uniform films are therefore capable, but not necessarily required, for high-performance WD in BPMs. The regions without WD catalyst coverage are likely inactive, as BPMs without WD catalysts require high voltages to pass current. Below we focus on data obtained from spray-coating (unless specified), as this method is amenable to large-area processing and manufacturing.

The U-shaped dependence of voltage on loading (Fig. 2b) might be explained by the ionic resistance of the WD catalyst layer. The WD catalyst layer is composed of solid nanoparticles and liquid water. The ions must move in the liquid phase or by hopping across the particle surfaces. Nanoparticle surfaces are the putative active sites for WD, which generates ionic carriers, so increasing the loading would be expected to improve performance. However, as the thickness and packing density of the WD catalyst layer increase, the transport lengths for OH^−^ and H^+^ also increase leading to an expected increasing ohmic loss. The nanoparticles, however, also likely provide H^+^ or OH^−^ from surface acid/base groups, therefore increasing the equilibrium ionic carrier concentration compared to pure water.

**Fig. 4:**
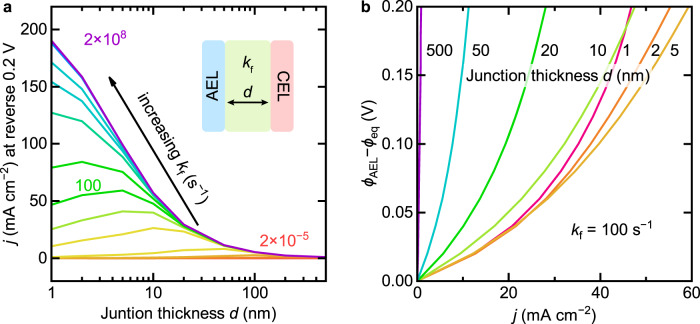
Steady-state numerical simulated results of BPMs with different junction thickness and WD rate constant. **a** Current density at reverse bias of 0.2 V as a function of junction thickness for different WD rate constants in the junction. **b** Polarization curves in reverse bias for different junction thickness with WD rate constant *k*_f_ = 100 s^−1^. Similar results using the reported diffusion coefficients for H^+^ an OH^−^ along with the fixed ion concentration in the membranes estimated based on the manufacturer specifications are in Supplementary Fig. 4. See Methods for more information.

To assess this behavior, we modified our BPM simulation. We kept the ionization equillibrium constant of water *K*_w_ the same as in bulk water, but increased both WD rate constant *k*_f_ and H^+^/OH^−^ recombination rate constant *k*_r_ (i.e., modeling a pure catalytic effect, with no change to the thermodynamics of the reaction). We simulate the total current density *j* at different voltages across the membrane as a function of distance between the AEL and CEL. The resulting current density as a function of junction thickness *d* (i.e., AEL-CEL distance) peaks, consistent with the experimental results (Fig. 4a). With higher WD rate constants, the optimal thickness is smaller and the peak current is higher. Based on the model, better WD catalysts provide higher currents at lower loading where ohmic losses are minimized.

This model, however, has limitations. The simulated reverse-bias polarization curves show that conductance (*dj*/*dV*) decreases with increased applied voltage. Further, a limiting current density in reverse bias is evident when the junction thickness and WD rate constant are small (Fig. 4b). The experimental results, however, show that the reverse-bias conductance increases with potential and approaches a constant (linear *j*-*V* response), and no limiting current density is apparent. This discrepancy suggests that the WD catalytic effect described by a constant, *η*_wd_-independent rate constant is insufficient to explain the enhanced WD in BPMs. Further, the best WD catalyst thickness in our experiments is 200–600 nm, but with this thickness, the model cannot produce the high current densities observed in experiments, even with a large WD rate constant (see the curve of *k*_f_ = 2 × 10^8 ^s^−1^ in Fig. 4a, for comparison, in bulk water *k*_f_ = 2 × 10^−5^ s^−1^). Previous simulation studies proposing WD driven by the second Wien effect used small junction thicknesses of usually <10 nm, leading to large interfacial electric field, and assumed an electric-field-dependent WD rate constant to generate curves that roughly match experiment^29–31^.

### Impedance analysis to isolate WD kinetics

**Fig. 5:**
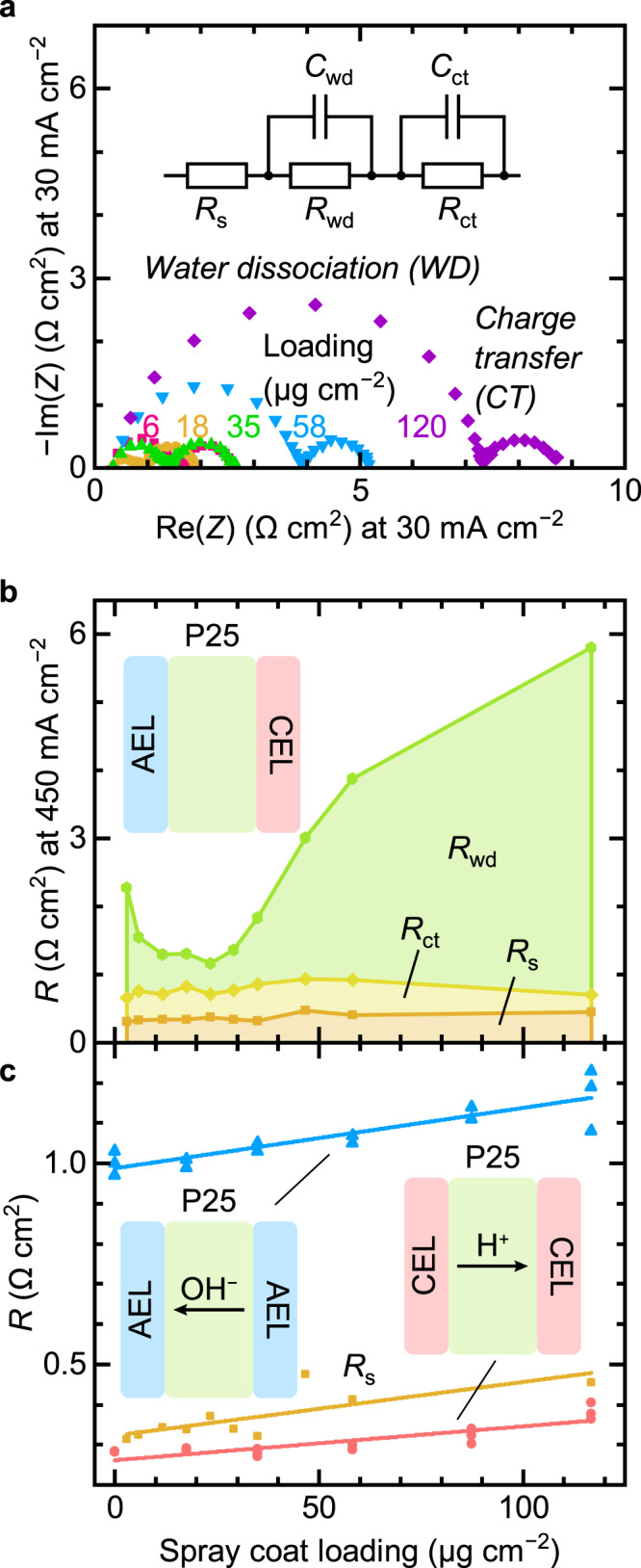
Impedance analysis of BPM electrolyzers. **a** Nyquist plots of BPM electrolyzers at 30 mA cm^−2^ with different loadings of TiO_2_-P25 deposited by spray coating WD catalysts. The high frequency semicircle is assigned to WD, while the low frequency one to charge transfer. The inset shows the equivalent circuit used to fit the EIS data. **b** Extracted resistance values at 450 mA cm^−2^ as a function of TiO_2_-P25 loading. **c** Comparison of series resistance *R*_s_ (orange) extracted from BPM electrolyzer EIS data at 450 mA cm^−2^ with the resistance of PEM (red) and AEM (blue) electrolyzers at 300–500 mA cm^−2^ as a function of TiO_2_-P25 loading.

To inform simulations and obtain quantitative information on the various charge-transfer, transport, and WD impedances of the BPM electrolyzer, we used electrochemical impedance spectroscopy (EIS)^7,32^. A typical Nyquist plot at 30 mA cm^−2^ with various TiO_2_-P25 loadings shows two semicircles (Fig. 5a). The lower frequency semicircle (right) is independent of the WD catalyst loading, while the one at higher frequency (left) is not. We keep the anode and cathode the same, so the CT impedances should be independent of WD catalyst loading. Thus, we associate the high-frequency semicircle with WD in the BPM, and the low-frequency one with the electrode CT processes. Similar trends are observed at other current densities (Supplementary Fig. 5). We construct an equivalent circuit (Fig. 5a) composed of a series resistance (*R*_s_) and two parallel *RC* circuits, to describe WD (*R*_wd_) and charge-transfer (*R*_ct_) accordingly, connected in series. We fit the impedance spectra using this equivalent circuit at 450 mA cm^−2^ (Fig. 5b, and Supplementary Fig. 6), and find that *R*_wd_ dominates the total resistance and is directly correlated with the cell voltage (Fig. 2b). *R*_ct_ is essentially independent with the WD catalyst loading, as expected. *R*_s_ increases slightly with increasing loading (see below). Therefore, the linear increase of total voltage with current observed at higher currents in Fig. 2a can be assigned largely to WD. However, from our equivalent-circuit model and impedance data, it is not clear whether the ionic resistance of the WD catalyst layer will be represented in *R*_s_ or *R*_wd_.

The EIS data can also be used to estimate the WD overpotential *η*_wd_ and compare with reported values. To compare with an industry-standard Neosepta BPM (~1.2 V in H-cell at 100 mA cm^−2^ and 30 °C, ${\eta }_{{{{{{\rm{wd}}}}}}}\approx 1.2{{{{{\rm{V}}}}}}-0.83{{{{{\rm{V}}}}}}=0.37{{{{{\rm{V}}}}}}$)^33^, we tested the BPM electrolyzer with optimal TiO_2_-P25 loading (~18 μg cm^−2^) at 30 °C (Supplementary Fig. 7 and Supplementary Discussion). EIS analysis shows that *R*_wd_ decreases from ~0.96 Ω cm^2^ to ~0.66 Ω cm^2^ as current increases from 5 mA cm^−2^ to 500 mA cm^−2^ and we calculate ${\eta }_{{{{{{\rm{wd}}}}}}}={\int }_{0}^{j}{R}_{{{{{{\rm{wd}}}}}}}\left(j\right){{{{{\rm{d}}}}}}j$. At 100 mA cm^−2^_,_ ${\eta }_{{{{{{\rm{wd}}}}}}}$ is 0.09 V, four times lower than Neosepta. The performance of other BPMs are compiled in literature^1,8^. Most of the membrane voltages are well above 1 V at 100 mA cm^−2^ (*η*_wd_ > 0.2 V). Shen et al. used Al(OH)_3_ in an electrospun 3D BPM junction and found *η*_wd_ ~0.2 V (estimated from the onset of the polarization curve) at 100 mA cm^−2^ at 25 °C^34^. Chen et al. reported an *iR* free voltage of 1.5 V at 500 mA cm^−2^ at ~25 °C using graphene oxide WD catalyst^21^, which is *η*_wd_ ~0.7 V compared to *η*_wd_ ~0.38 V under the similar conditions for our systems. By increasing temperature, as in Fig. 2, here *η*_wd_ is substantially reduced to, e.g., only 0.24 V at 500 mA cm^−2^ and 55 °C. The systems we studied are nominally 2D BPMs and we focused specifically on the WD catalytic processes. The developments we report are orthogonal to the progress made in, for example, electrospinning of 3D-junction BPMs. We expect that if controlled WD catalyst layers like reported here can be integrated into 3D electrospun BPMs, further performance enhancements will be possible.

### Ion transport in the WD catalyst layer

To measure the ionic conductivity in the WD catalyst layer, we built proton-exchange-membrane (PEM) and anion-exchange-membrane (AEM) electrolyzers where the TiO_2_-P25 layers were sandwiched between either two identical CELs or AELs (Fig. 5c, and Supplementary Fig. 8). We measured electrolyzer polarization curves and fit the region from 300 to 500 mA cm^−2^ to a line to obtain the differential resistance. At these high currents, the differential resistance is dominated by ionic transport (both HER and OER rates increase exponentially with overpotential). Because only H^+^ transports through the TiO_2_-P25 layer in PEM electrolyzers and only OH^−^ through TiO_2_-P25 in AEM electrolyzers, we are able to measure the ionic conductivity of H^+^ and OH^−^ in the TiO_2_-P25 layer separately. As the TiO_2_-P25 WD catalyst loading increased from 0 to ~120 μg cm^−2^ (a ~2.4-μm-thick film) the resistance of PEM electrolyzers increases by only ~0.10 Ω cm^2^, while for AEM electrolyzers only ~0.17 Ω cm^2^. Based on this data, we estimate the ionic conductivity of the TiO_2_-P25 layer to be ~2.4 mS cm^−1^ for H^+^ and ~1.4 mS cm^−1^ for OH^−^ at 55 °C. Assuming equivalent conductivities of H^+^ and OH^−^ in the water/TiO_2_-P25 WD-catalyst layer as in pure water, and neglecting temperature and concentration effects, these results give an average concentration of H^+^ and OH^−^ in the TiO_2_-P25 layer of ~7 mmol L^−1^. In comparison, the conductivities of Nafion 212 and PAP-TP-85 at 60 °C are both over >50 mS cm^−1^ ^35,36^. From the impedance analysis, we find that the change of *R*_wd_ is ~5 Ω cm^2^ from ~18 to ~120 μg cm^−2^ (~360 nm to ~2.4 μm) but the change in the ionic resistance of the TiO_2_-P25 layer is only ~0.10 or ~0.17 Ω cm^2^. This demonstrates that a simple increase in ionic resistance due to the WD catalyst layer thickness cannot explain the decrease in BPM performance for WD catalyst loadings above the optimum. Interestingly, we also find that the increase in differential resistance in the reference AEM and CEM electrolyzers is comparable to the increase of *R*_s_ (~0.15 Ω cm^2^) measured by impedance in BPM electrolyzers as the WD catalyst loading is increased (Fig. 5c). This result indicates that the ionic resistance of the WD catalyst layer is represented as a component of *R*_s_ in the equivalent circuit and not *R*_wd_. This impedance data thus shows that it is possible to confidently separate the ionic transport, charge transfer, and WD resistances via EIS analysis, which is of significant value in optimizing systems.

### WD catalyst surface area

**Fig. 6:**
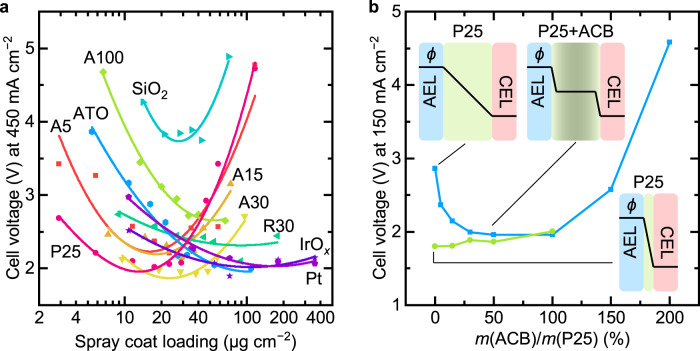
Performance of BPM electrolyzers with various WD catalysts. **a** Cell voltage of BPM electrolyzers as a function of spray-coated loading of various WD catalysts at 450 mA cm^−2^. Lines are added to serve as a guide for the eye. For various TiO_2_, A = anatase and R = rutile. The number denotes the size of the nanoparticles (nm) provided by the manufacture. ATO = Sb:SnO_2_. **b** Cell voltage of BPM electrolyzers as a function of the mass ratio of acetylene carbon black (ACB) and TiO_2_-P25 at 150 mA cm^−2^. The blue line is for a thick layer of ∼120 μg cm^−2^ (∼2.4 μm) TiO_2_-P25, and the green line is for a thin layer of TiO_2_-P25 at optimal loading ∼18 μg cm^−2^ (∼360 nm). Only one of each type of device was fabricated for the data in this figure to illustrate trends, except for TiO_2_-P25. The error was estimated to be less than 5% (one standard deviation) based on seven devices fabricated with the best loading of TiO_2_-P25 catalysts (Supplementary Fig. S3). Insets are schematic proposed electric-potential profiles across the BPM junction.

Previously, Oener et al. studied the WD activity of various metal-oxide nanoparticles with similar diameters^8^. One hypothesis is that smaller nanoparticles of the same metal oxide will have better WD performance because of higher specific surface area (SSA, see Methods and Supplementary Table 1 and Table 2). We studied the loading dependence with nominally 5, 15, 30, and 100 nm anatase and 30 nm rutile particles (Fig. 6a, and Supplementary Fig. 2). The 5, 15, and 30 nm anatase shows the U-shaped voltage response with loading, indicative of an optimal loading between 10 and 30 μg cm^−2^. The performance of 100-nm anatase and 30-nm rutile TiO_2_, however, continues to improve with loading to much higher values. If we compare the performance of each WD catalyst at its optimal loading, the 30 nm anatase and TiO_2_-P25 (around ~20–30 nm, ~80% anatase) are substantially better than the 5-nm anatase even though the 5-nm particles have a SSA seven times that the 30-nm ones (Supplementary Table 2). This surprising result contradicts typical behavior of heterogeneous catalysts where higher SSA yields higher activity.

### Field effects on water dissociation

These unexpected results led us to consider in more detail the second Wien effect (see Supplementary Discussion). According to Onsager’s theory, the WD rate increases nearly exponentially with the electric field. If the electric field is concentrated in some regions of the BPM junction, the overall WD rate might be higher than if the field is averaged across the whole junction. In fact, Chen et al. suggested that WD might be enhanced by using electronically conducting materials (see Supplementary Discussion)^20^. We studied the loading dependence of electronically insulating materials like SiO_2_, as well as conductive materials such as antimony-doped tin oxide (ATO), IrO_*x*_, and Pt (Fig. 6a and Supplementary Fig. 9). SiO_2_ is the worst WD catalyst. ATO, IrO_*x*_, and Pt all show good performance, but with much higher mass loading than anatase TiO_2_. We measure the apparent electronic conductivities of these nanoparticles (using a simple two-probe setup, see Methods) and correlated those with their performance in BPM electrolyzers (Supplementary Fig. 10). Better performance is generally observed for nanoparticles with higher electronic conductivity (although electronic conductivity is clearly not the only important parameter, e.g., the acid-base properties of the surface are also critical to the catalytic effect^8^, as is the loading, etc.).

Based on this data, we hypothesized that adding electronically conductive carbon to the TiO_2_-P25 would improve performance in situations where that WD catalyst layer was too thick – and thus the electric field is too small with the electrochemical potential drop smeared out across the junction – by concentrating the electrochemical potential drops and electric field to the interfacial regions. We added different amounts of acetylene carbon black (ACB) nanoparticles, an electronic conductor, to BPMs with the most TiO_2_-P25 (~120 μg cm^−2^, ~2.4 μm thick) and measured BPM electrolyzer performance (Fig. 6b, blue curve, and Supplementary Fig. 11). The WD performance is substantially improved after adding ACB at a mass ratio near 1:1. We also tested ACB and TiO_2_-P25 mixtures at the previously determined optimal loading (~18 μg cm^−2^ and ~360 nm). For these thinner WD catalyst films, no performance improvement was observed (Fig. 6b, green curve, and Supplementary Fig. 11). ACB alone was a poor WD catalyst (Supplementary Fig. 12). These experimental results are consistent with the mobile electrons in the conductive WD catalyst polarizing the WD catalyst layer in response to the net electric potential drop across the junction, with a positive electronic charge on the junction side facing the CEM and negative electronic charge on the AEM side. As a result, the electric potential drop is focused into a narrow region at the AEL|WD-catalyst and WD-catalyst|CEL interfaces. Based on Onsager’s theory, the resulting increased field would increase the rate of WD almost exponentially in that region. The excess WD catalyst in the middle of the WD catalyst film then simply adds a series resistance (shown to be small by the ionic conductivity experiments above). For optimally thin WD catalyst, however, the electric field is apparently already sufficiently strong that the addition of ACB reduces performance (e.g., by reducing density of WD active sites).

### Stability

We tested the stability of the BPM electrolyzer with the best loading (~18 μg cm^−2^) of TiO_2_-P25 at 500 mA cm^−2^ for 36 h at 55 °C (Supplementary Fig. 13). The voltage was found to increase at ~15 mV h^−1^ for the first 18 h and at ~6 mV h^−1^ after that, comparable to what we observed in AEM electrolyzers^37^. This data suggests a similar degradation mechanism, i.e., ionomer degradation at the alkaline anode evolving O_2_. In the Nyquist plots collected at 30 mA cm^−2^ every 6 h, the initial two semicircles evolved into three semicircles. Equivalent circuit fits show that *R*_s_ is constant and *R*_wd_ slightly increased (~1.7 mΩ cm^2^ h^−1^) over 36 h, indicating good stability of the water-dissociating BPM junction. In contrast, the total charge-transfer resistance *R*_ct1_ + *R*_ct2_ increased from ~1.1 Ω cm^2^ to ~3.3 Ω cm^2^, consistent with the expected anode degradation.

## Discussion

We elucidated the key roles of electronic and ionic conductivity of the WD catalyst within the BPM junction enabling record BPM-electrolyzer performance with pure-water feed. For semiconducting WD catalysts such as TiO_2_, there is an optimal range of loading/thickness, 10-30 μg cm^−2^ (200-600 nm in thickness), while for electronic conductors, the optimal is higher and the range larger. Reference measurements in proton-exchange-membrane and anion-exchange-membrane electrolyzers show that, surprisingly, the ionic resistance of the WD catalyst layer is relatively unimportant, even for TiO_2_ films ~2 µm in thickness. Combining conductive carbon nanoparticles with thick TiO_2_ WD catalyst layers dramatically improves performance compared to either component in isolation, apparently by focusing the junction electric field. Impedance analysis enables clear separation of ionic transport, anode/cathode, and WD resistances which, taken togethor, supports these conclusions. These data show that WD catalysts operate via mechanisms that are more complex in high-electric-field BPM junctions compared to conventional heterogeneous chemical catalysts. Not only do intrinsic surface activities matter – probably governed by acid/base and surface-chemical properties – but properties like the dielectric constant and electrical conductivity of the WD catalyst play a key role in affecting interfacial junction physics and WD rate.

Additional work is needed to fully understand the WD mechanism in BPMs. Particularly useful would be experiments to map the concentration profiles of H^+^ and OH^−^ and the electric field within the BPM junction and correlate this to WD rates. Measuring such profiles for WD catalysts of different compositions and layering schemes would be useful to understand the molecular details of the catalysis process, including the specific chemical sites where WD occurs. Studying the reverse reaction, H^+^ and OH^−^ recombination, and whether that process can also be catalyzed following the same mechanistic principles as WD is also of fundamental interest and important for new BPM applications. Such advances are central for the design of optimized WD catalysts and BPMs based on the electronic, ionic, surface-chemical, transport, and other materials properties. Finally, the high current density and low resistance of the BPM electrolyzers reported here using only earth-abundant WD catalysts are impressive in their own right. This will be likely to benefit the applications highlighted in the introduction, among others. Further improvements are expected by combining optimized catalytic layers with 3D interpenetrating BPM junctions developed by others^34^.

## Methods

### Cell fabrication and measurements

The gas diffusion layers (GDLs) are fabricated by spray coating. The anode ink is prepared in a 20 mL scintillation vial with 0.1 g IrO_*x*_ (Pajarito Powder) or core/shell Ir/IrO_*x*_ (Fuel Cell Store), 0.5 g H_2_O, 1.7 g isopropyl alcohol (IPA), 0.1 g PiperION-A5 ionomer suspension (TP-85, 5% w/w, Versogen). The ink is sonicated until the nanoparticles are well dispersed. The substrate for the anode GDL is stainless steel 25AL3 (Bekaert Bekipor^®^). The substrate is cut into a square of 5 cm × 5 cm and taped on a hot plate of 90 °C. Two vials of the ink are spray coated on the substrate. The loading of the catalyst is ~2 mg cm^−2^. Then PiperION-A5 sonomer suspension is sprayed on top of the catalyst layer until the mass of the ionomer reaches 10–20% the catalyst mass. Finally, the GDL is cut into squares of 1.0 cm × 1.0 cm for later use. For PEM electrolyzers, platinized Ti-fibre felt (Fuel Cell Store) is used as substrate instead of stainless steel to prevent corrosion under acidic and anodic conditions.

The cathode ink is prepared in a 20 mL scintillation vial with 0.1 g Pt black (high surface area, Fuel Cell Store), 1.5 g H_2_O, 1.7 g IPA, 0.1 g D520 Nafion™ Dispersion (alcohol-based 1000 EW at 5 wt%, Fuel Cell Store). The ink is sonicated until the nanoparticles are well dispersed. The substrate for the GDL is Toray Carbon Paper 090 (value pack, wet proofed, Fuel Cell Store). The substrate is cut into a square of 5 cm × 5 cm and taped on a hot plate of 90 °C. Two vials of the ink are spray coated on the substrate. The loading of the catalyst is ~2 mg cm^−2^. Then D520 Nafion™ dispersion is sprayed on top of the catalyst layer until the mass of the ionomer reaches 10–20% the catalyst mass. The GDL is the cut into squares of 1.0 cm × 1.0 cm for later use.

PiperION-A40-HCO3 (TP-85, 40 μm, Versogen) is used as the anion-exchange layer (AEL). The membranes are pre-treated according to the manufacturer instructions. The AEL is soaked in 0.5 M KOH for >1 h, stored in fresh 0.5 M KOH, and rinsed in ultra-pure H_2_O before use. Nafion™ 212 (Fuel Cell Store) is used as the cation exchange layer (CEL). According to the manufacturer, the membrane comes in a pre-protonated state and does not need additional pre-treatment. Thus, the CELs are soaked and stored in H_2_O. Both membranes are cut into squares 1.5 cm × 1.5 cm before use.

The measured and manufacturer-provided properties of all the WD catalyst nanoparticles studied are listed in Supplementary Tables 1 and 2. These nanoparticles were spray coated from an ink onto the CEL. A mother ink of 2 wt% is prepared in H_2_O and sonicated until the nanoparticles are well dispersed. The ink for spray coating is made by diluting this mother ink. Different masses of the mother ink are transferred to a 20 mL scintillation vial. H_2_O is added until the total mass reaches 0.5 g, and then 1.7 g IPA is added. For high loading of IrO_*x*_ and Pt, the amount of H_2_O and IPA is increased to aid dispersion. This diluted ink is sonicated until the nanoparticles are well dispersed before spray coating. The Nafion membrane is cut into a square of 1.5 cm × 1.5 cm and taped in the bottom of a glass petri dish so that the exposed area is 1.2 cm × 1.2 cm. Then the petri dish is placed on a hot plate at 90 °C. The diluted ink is spray coated onto the CEL. To improve uniformity, the petri dish is rotated 90° every 10 spray bursts. After the spray coating, the petri dish is removed from the hot plate, and the tape is removed carefully to prevent damage to the CEL. H_2_O is added around the CEL so that it absorbs water and delaminates from the petri dish. The coated CEL is transferred to a container with H_2_O before use.

For the spin-coated samples, a mother ink of 10 wt% TiO_2_-P25 in water/IPA mixture (1:1 by weight) is prepared. The mother ink is horn sonicated for 10 min and filtered through a 5 μm syringe filter to remove larger aggregates (Acrodisc^®^ 32 mm syringe filter with 5 μm Supor^®^ membrane). The mother ink is diluted with water/IPA mixture (1:1 by weight) to make the spin coating inks of the concentrations indicated. The CEL is cut into a square of 1.5 cm × 1.5 cm and its edges are taped on a glass slide for spin coating. Drops of the ink are added on the membrane until it is fully covered. The membrane is then spun at 3000 rpm for 30 s. The 2.0 wt% sample is made by spin coating two layers of the 1.0 wt% ink.

The electrolyzer was built from PEM fuel-cell hardware (Fuel Cell Store). A homemade stainless-steel flow field was used instead of the original graphite anode. The gaskets used in the assembly have an active area of 1.0 cm × 1.0 cm. To assemble the electrolyzer, several gaskets with a total thickness of 0.032″ are placed on top of the cathode flow field, and one Ti spacer (sintered Ti frits electroplated with 1 μm Pt, 1 cm × 1 cm, Baoji Yinggao Metal Materials Co., Ltd.) is placed in the square hole of the gasket, followed by the cathode GDL, with the HER catalyst side facing up. The BPM is then placed on top of the cathode GDL with WD catalyst sandwiched between the CEL and AEL. The CEL is in contact with the cathode GDL. After that, several gaskets with a total thickness of 0.037″ are placed around the BPM. The anode GDL is then placed in the square hole of the gasket with the OER catalyst facing the AEL. A second Ti frit spacer is then placed on top of the anode GDL. Finally, the anode flow field and current collector is bolted together to seal the system. The bolts are tightened by a torque wrench to 50 inch-pounds. Pure de-ionized H_2_O heated at 60 °C is fed to both the anode and cathode. The electrochemical tests are started after the cell hardware reaches the equilibrium temperature (55 ± 2 °C, maximum fluctuation) using a BioLogic VSP-300 potentiostat.

The current density is first held at 10 mA cm^−2^ for 1 min, then stepped from 50 to 500 mA cm^−2^ with 50 mA cm^−2^ steps (1 min each step), and finally held at 500 mA cm^−2^ for 10 min. For the BPMs with ACB, a larger voltage was observed during the initial steps of increasing current density, so the current steps were maintained for 1–70 min instead. Finally, the current density was stepped down in the reverse order and the voltage at each step was measured. The reported polarization curves are generated from the average voltages measured over the last second of each current step. The current is then stepped up again (10 s each step and then held at 500 mA cm^−2^ for 1 min) to prepare for electrochemical impedance spectroscopy (EIS). Impedance data are recorded at each current density step from 500 mA cm^−2^ down to 50 mA cm^−2^ with 50 mA cm^−2^ steps, as well as current densities of 40, 30, 20, 10 and 5 mA cm^−2^. An AC amplitude of 6% of the applied DC current density is used from 500 to 20 mA cm^−2^. For 10 and 5 mA cm^−2^, the AC amplitude is 1 mA cm^−2^. The frequency was scanned from 600 kHz to 20 mHz with 4 points per decade. Most of the EIS data are fitted using Bio-Logic EC-Lab V11.33. The stability EIS data where fit using impedance.py^38^.

To support the formation of H_2_ and O_2_ in a 2:1 ratio and show near-unity Faradaic efficiency, we measured the amount of evolved gas with graduated cylinders at room temperature (22 °C, Supplementary Fig. 14). Before passing current, two graduated cylinders (50 mL for O_2_ and 100 mL for H_2_) are filled with water and placed upside down in the water tank (total volume of water is ~5.5 L). The gas bubbles are generated at the electrodes under high local supersaturation during electrolysis and carried by the water flow (~100 mL min^−1^ for the anode and ~60 mL min^−1^ for the cathode) for collection in the inverted graduated cylinders in about 10 s. After the electrolysis, the volume of the gas is read by leveling the water inside the graduated cylinders with the water in the tank. We applied 500 mA for 20 min, and the theoretical volumes of H_2_ and O_2_ are 75.30 mL and 37.65 mL, while the experimental values are 75.5 ± 0.5 mL and 37.0 ± 0.5 mL (uncertainty of the graduated cylinder). Thus, the Faradaic efficiency is ~100% for H_2_ and ~98% for O_2_. Given the short transit time between bubble detachment at the electrode and collection in the cylinder, little O_2_, and negligible H_2_, is apparently lost due to dissolution in the recirculating water. The smaller O_2_ Faradaic efficiency might also be due in part to oxidation of ionomer, as we have discussed and shown to limit durability of current alkaline-membrane pure-water electrolysis systems^37^. SEM images of a BPM after testing in the electrolyzer are shown in Supplementary Fig. 15. No evidence for membrane breaking or cracking is observed.

### Quantifying the loading and thickness of WD catalysts

Microscope cover-glass slides were used as substrates to quantify the loading and thickness of TiO_2_-P25 because the membrane mass is highly sensitive to water content and changes over the course of the measurement and processing. The ink is spray coated on the same sized cover glass as the membrane (1.2 × 1.2 cm^2^) and the mass change is measured using a semi-microbalance (Sartorius Quintix™, see Supplementary Fig. 16). We found that the rate of TiO_2_-P25 WD catalyst deposition was 27 ± 3 μg cm^−2^ (standard error of fitting) per mg of ink in the spray-coating solution. The thickness is then determined by cross-sectional environmental scanning electron microscopy (ESEM, pressure of 40 Pa in H_2_O) and energy-dispersive X-ray spectroscopy (EDS) mapping (ThermoFisher Apreo 2S). The EDS signal of Ti is integrated and plotted as a function of position. A Gaussian function is used to fit the curve and the full width at half maximum (FWHM) is used to represent the thickness (Supplementary Fig. 16). We found that the films were 0.59 ± 0.09 μm (standard error of fitting) in thickness per mg of TiO_2_-P25 WD catalyst in the ink. We thus conclude that for the best performance case (0.6 mg TiO_2_-P25 in ink), the WD catalyst loading is ~18 μg cm^−2^ and the thickness is ~360 nm. The loadings of other WD catalysts are determined using a similar calibration method. The BPM cross-section samples are prepared by immobilization (LR White resin) and microtoming.

### N_2_ adsorption-desorption experiments

Nitrogen (N_2_) adsorption/desorption isotherms were obtained at 77 K using Micromeritics ASAP 2020 surface area analyser. Specific surface areas (SSA) of the samples were calculated using Brunauer–Emmett–Teller (BET), while the pore volume (*V*_p_) was calculated using the Barrett, Joyner, and Halenda (BJH) adsorption curves. Before measurements, the TiO_2_ nanoparticles were dispersed in hexanes and dried at room temperature under vacuum for 18 h. Prior to analysis, the samples were activated at 423 K for at least 24 h to remove the solvent and trapped gas. Activation was considered complete when the outgassing rate fell below 2.5 μtorr min^−1^. The sample mass was determined by the difference in mass between the empty sample tube and the loaded sample tube post-activation. Based on IUPAC classification, all TiO_2_ nanoparticles showed type-III isotherms, which are indicative of macroporous materials. In all samples, a type H_3_ hysteresis was observed demonstrating macroporosity with narrow slit-like pores^39^. BET surface areas and calculated pore volumes are given in Supplementary Table 2.

### Electronic conductivity

The apparent electronic conductivities of WD catalysts are measured using a simple two-electrode setup. A pellet of the nanoparticles is made with a die and press (Quick Press Sigma-Aldrich^®^) and a homemade polyether-ether-ketone (PEEK) collar. Then the metal plungers on the press are used as the two electrical contacts to measure the current-voltage response of the compressed powder pellet. A polarization curve is collected between ±0.1 V at 5 mV s^−1^. The curve is fitted to a line to extract the apparent electronic conductance $G$. The apparent electronic conductivity $\kappa$ is calculated by $\kappa =\frac{Gl}{A}$, where *G* is the conductance extracted from the current-voltage response, $l$ is the thickness of the pellet determined by the difference of the length of the die set with and without the nanoparticles measured by a caliper, $A=0.4\,{{{{{{\rm{cm}}}}}}}^{2}$ is the area of the pellet.

### Numerical simulations

The BPM model was built in COMSOL Multiphysics® 5.5 with only two mobile ions, H^+^ and OH^−^, consistent with the BPM electrolyzer devices. The simulation model is built with the least-possible components, including reaction (catalytic and non-catalytic), transport (diffusion and migration), and the physical dimensions of the system such as the junction thickness. The purpose of the model is *i*) to illustrate the underlying fundamental physics of how the various potentials develop under operation to provide a framework for understanding how the introduction of catalytic materials with different dielectric properties can modulate this picture, and *ii*) to illustrate the fundamental trade-off between ionic resistance and catalyst loading in the junction in the context of the experimental data. It would be straightforward to increase the complexity of the model to include a series of chemical reaction steps for the catalysts, hypothesized electric-field effects explicitly, variable surface charge on the catalyst particles, etc. Yet doing so would not provide new insight and would likely make the model less useful due to the large numbers of adjustable parameters which are not known based on experiment. Regardless of the mechanistic details, the net result is that in reverse bias H_2_O is dissociated into H^+^ and OH^−^. We thus write ${k}_{{{{{{\rm{f}}}}}}}$ as the net forward WD rate constant, and ${k}_{{{{{{\rm{r}}}}}}}$ as the net reverse (recombination) rate constant. For simplicity, we treat activity coefficients as unity and use concentrations for the equilibrium constant. At equilibrium,

1 $$\begin{array}{c}{K}_{{{{{{\rm{eq}}}}}}}=\frac{{c}_{{{{{{{\rm{H}}}}}}}^{+}}{c}_{{{{{{{\rm{OH}}}}}}}^{-}}}{{c}_{{{{{{{\rm{H}}}}}}}_{2}{{{{{\rm{O}}}}}}}c^\circ }=\frac{{K}_{{{{{{\rm{w}}}}}}}c^\circ }{{c}_{{{{{{{\rm{H}}}}}}}_{2}{{{{{\rm{O}}}}}}}}=\frac{{k}_{{{{{{\rm{f}}}}}}}}{{k}_{{{{{{\rm{r}}}}}}}c^\circ }\end{array}$$

where ${K}_{{{{{{\rm{eq}}}}}}}$ is the equilibrium constant, ${c}_{i}$ is the molar concentration of species $i$, $c^\circ =1\;{{{{{\rm{mol}}}}}}\;{{{{{{\rm{L}}}}}}}^{-1}$is the reference molar concentration, and ${K}_{{{{{{\rm{w}}}}}}}$ is the ionization constant of water (${10}^{-14}\;{{{{{\rm{at}}}}}}\;25^{\,\circ}{{{\rm{C}}}}$). We take ${c}_{{{{{{{\rm{H}}}}}}}_{2}{{{{{\rm{O}}}}}}}=55.6\;{{{{{\rm{mol}}}}}}\;{{{{{{\rm{L}}}}}}}^{-1}$ as a constant. ${k}_{{{{{{\rm{r}}}}}}}$ has been determined experimentally in pure water using a high-voltage impulse:^40^ *k*_r_ = (1.3 ± 0.2) × 10^11^ L mol^−1^ s^−1^(at 25 °C). Therefore, the WD rate constant can be calculated (in bulk, free water) as

2 $$\begin{array}{c}{k}_{{{{{{\rm{f}}}}}}}=\frac{{K}_{{{{{{\rm{w}}}}}}}{k}_{{{{{{\rm{r}}}}}}}{\left(c^\circ \right)}^{2}}{{c}_{{{{{{{\rm{H}}}}}}}_{2}{{{{{\rm{O}}}}}}}}\approx 2\times {10}^{-5}\,{{{{{{\rm{s}}}}}}}^{-1}\left({{{{{\rm{at}}}}}}\,25^{\,\circ}{{{\rm{C}}}}\right)\end{array}$$

We built a 1-D geometry composed of three consecutive intervals, representing the AEL, the WD catalyst layer (junction), and the CEL accordingly from left to right (Fig. 1b). The thicknesses of AEL and CEL are both fixed to be 50 μm, while the thickness of the junction $d$ was varied. Transport of species $i$ is driven by the gradient in electrochemical potential ${\bar{\mu }}_{i}$. The continuity equation (mass balance) at steady state requires that ${{{{{\boldsymbol{\nabla }}}}}}\cdot {{{{{{\boldsymbol{J}}}}}}}_{i}={R}_{i}$, where ${{{{{{\boldsymbol{J}}}}}}}_{i}$ is the flux, and ${R}_{i}$ production rate of species $i$, calculated by

3 $$\begin{array}{c}{R}_{{{{{{{\rm{H}}}}}}}^{+}}={R}_{{{{{{{\rm{OH}}}}}}}^{-}}={k}_{{{{{{\rm{f}}}}}}}{c}_{{{{{{{\rm{H}}}}}}}_{2}{{{{{\rm{O}}}}}}}-{k}_{{{{{{\rm{r}}}}}}}{c}_{{{{{{{\rm{H}}}}}}}^{+}}{c}_{{{{{{{\rm{OH}}}}}}}^{-}}\end{array}$$

Ignoring concentrated electrolyte effects, ${{{{{{\boldsymbol{J}}}}}}}_{i}$ is given by the Nernst–Planck equation (without convection)

4 $$\begin{array}{c}{{{{{{\boldsymbol{J}}}}}}}_{i}=-\frac{{{c}_{i}D}_{i}}{{RT}}{{{{{\boldsymbol{\nabla }}}}}}{\bar{\mu }}_{i}=-{D}_{i}{{{{{\boldsymbol{\nabla }}}}}}{c}_{i}-\frac{{{c}_{i}D}_{i}{z}_{i}F}{{RT}}{{{{{\boldsymbol{\nabla }}}}}}\phi \end{array}$$

where ${D}_{i}$ is the diffusion coefficient (assumed to be ${10}^{-4}\,{{{{{\rm{c}}}}}}{{{{{{\rm{m}}}}}}}^{2}\,{{{{{{\rm{s}}}}}}}^{-1}$ for both H^+^ and OH^−^ for simplicity, while in Supplementary Fig. 4, ${D}_{{{{{{{\rm{H}}}}}}}^{+}}=9.311\times {10}^{-5}\,{{{{{\rm{c}}}}}}{{{{{{\rm{m}}}}}}}^{2}\,{{{{{{\rm{s}}}}}}}^{-1}$, and ${D}_{{{{{{{\rm{OH}}}}}}}^{-}}=5.273\times {10}^{-5}\,{{{{{\rm{c}}}}}}{{{{{{\rm{m}}}}}}}^{2}\,{{{{{{\rm{s}}}}}}}^{-1}$^41^), ${z}_{i}$ is the charge number, $\phi$ is the electric potential. $F$, $R$, and $T$ denote Faraday’s constant, the gas constant, and temperature respectively. Poisson’s equation is used to couple the charged species with electric potential

5 $$\begin{array}{c}{{{{{{\boldsymbol{\nabla }}}}}}}^{2}\phi =-\frac{F\left[\sum {c}_{+}-\sum {c}_{-}\right]}{{\varepsilon }_{0}{\varepsilon }_{{{{{{\rm{r}}}}}}}}\end{array}$$

where ${c}_{+}$ is the molar concentration of positive charges (e.g., H^+^, and fixed charges in AEL, set to be 1 mol L^−1^, while in Supplementary Fig. 4 set to be 2.4 mol L^−1^ ^35^), and ${c}_{-}$ is the molar concentration of negative charges (e.g., OH^−^, and fixed charges in CEL, also set to be 1 mol L^−1^, while in Supplementary Fig. 4 set to be 1.8 mol L^−1^ ^31^). ${\varepsilon }_{0}$ is the vacuum permittivity and ${\varepsilon }_{{{{{{\rm{r}}}}}}}=78$ is the relative dielectric constant of bulk water. For the outer boundary of the CEL, we assume *c*_OH_− = ${10}^{-14}$mol L^−1^, ${c}_{{{{{{{\rm{OH}}}}}}}^{+}}=$ 1 mol L^−1^ (in Supplementary Fig. 4${c}_{{{{{{{\rm{OH}}}}}}}^{-}}$ = 10^−14^/1.8 mol L^−1^, *c*_H_+ = 1.8 mol L^−1^), and set $\phi =0\,{{{\rm{V}}}}$. For the outer boundary of the AEL, we assume *c*_OH_− = 1 mol L^−1^, *c*_H_+ $=$ 10^−14^ mol L^−1^ (in Supplementary Fig. 4*c*_OH_− = 2.4 mol L^−1^, *c*_H_+ $=$ 10^−14^/2.4 mol L^−1^), and $\phi$ is variable. At equilibrium, $\phi$ at the outer boundary of AEL can be derived by equating ${\bar{\mu }}_{i}$ at two boundaries:

6 $$\begin{array}{c}{\phi }_{{{{{{\rm{eq}}}}}}}=\frac{{RT}}{{z}_{i}F}{{{{{\rm{ln}}}}}}\frac{{c}_{i}\left({{{{{\rm{CEL}}}}}}\right)}{{c}_{i}\left({{{{{\rm{AEL}}}}}}\right)}\approx 0.83{{{{{\rm{V}}}}}}\left({{{{{\rm{at}}}}}}\,25^\circ {{{{{\rm{C}}}}}}\right)\end{array}$$

whether calculated by H^+^ or OH^−^.

The mesh was defined by the maximum element size with denser elements at the interfaces. In AEL and CEL it is 1000 times smaller than the membrane thickness. In the junction, it is 100 times smaller than the junction thickness. At the WD-catalyst|AEL and CEL|WD-catalyst interfaces it is 10,000 times smaller than the junction thickness.

## Supplementary information

Supplementary information

## Data Availability

The data generated in this study have been deposited in the Science Data Bank (https://www.scidb.cn/en) with 10.57760/sciencedb.01825.

## Code availability

The COMSOL simulation files are available upon request from the authors.
