# Supplementary material for: Design principles for water dissociation catalysts in high-performance bipolar membranes
Source: Nat Commun. 2022 Jul 4;13:3846. doi: 10.1038/s41467-022-31429-7 (PMC9253156; doi:10.1038/s41467-022-31429-7)
Supplement: Supplementary file 1 — Supplementary information [file 41467_2022_31429_MOESM1_ESM.pdf]

Supplementary Information:

Design Principles for Water Dissociation Catalysts in High-Performance Bipolar Membranes

Lihaokun Chen[1], Qiucheng Xu[1,2], Sebastian Z. Oener[1,3], Kevin Fabrizio[1], Shannon W. Boettcher[1*]

[1] Department of Chemistry and the Oregon Center for Electrochemistry, University of Oregon, Eugene, Oregon 97403, United States

[2] Surface Physics and Catalysis (Surf Cat) Section, Department of Physics, Technical University of Denmark, 2800 Kgs. Lyngby, Denmark

[3] Present: Department of Interface Science, Fritz Haber Institute of the Max Planck Society, 14195 Berlin, Germany

To whom correspondence should be addressed:

*swb@uoregon.edu

Supplementary Discussion

Complexity of the BPM junction. The BPM junction is a complex system and difficult to study under operating conditions as it relies on the function of buried interface(s) inside the BPM and is usually operated in aqueous electrolytes. Electrochemical impedance and current-voltage analysis is the simplest way to characterize a BPM junction. Conventional BPMs electrochemical tests are carried out in H-cells with soluble supporting electrolytes[1], where salt ions other than H^+ and OH^- also contribute to the current. Due to the non-ideal selectivity of the AEL and CEL, "co-ion" transport complicates the analysis. To circumvent these problems, we have adopted the electrolyzer setup with pure-water feed and no co-ions[2].

Our previous work[2] showed a correlation between the point of zero charge (PZC) of oxide nanoparticles used as bilayer catalysts in the junction, the local pH, and the WD activity. The best systems had basic oxides such as NiO in contact with the AEL and acidic oxides such as IrO_2 in contact with the CEL. The bilayer system, although mechanistically and practically interesting in the context of controlling oxide chemistry for a specific local pH, increases the complexity and is not well-suited for careful studies of the interface BPM physics such as the electric field distribution and electrical/ionic conductivity in the junction. Further, the molecular details of WD remain unclear, particularly for metal nanoparticles where PZC is not a particularly useful concept due to the lack of substantial proton adsorption. The metals may catalyze WD instead through dissociative adsorption ($H_2O \rightarrow H_{ad} + OH_{ad}$) and desorption, and the electronic conductivity may also be important. In the previous work we also only roughly controlled and studied the effects of WD catalyst loading[2]. In some cases, small amounts of ionomer were added to aid the dispersion of the WD-catalyst ink, which further increases BPM junction complexity. The complications led us to return to well-defined single-layer systems, avoid using ionomer in the junction, and work to make the films as uniform as possible. We expect the new physical insight gathered here will however also be applicable to more-complex multilayer WD-catalyst interfaces.

The second Wien effect. The original experiment that led to the discovery of the second Wien effect was quite different from a BPM. The second Wien effect was discovered by measuring the resistance change of an electrolyte in a cell under high electric field (up to $\sim 2\times10^7$ V m^{-1}), generated by discharge through a spark gap[3-6]. The time constant of the circuit is $\sim 10^{-5}$ s or less. The equivalent ionic conductivity of weak electrolytes increased with the electric field and it was proposed to be due to increased dissociation of weak electrolytes. Later Onsager developed a quantitative explanation of this phenomenon and derived an equation to describe the relationship between dissociation constant and electric field[7].

Although the second Wien effect has been used to explain enhanced weak electrolyte dissociation for decades, it is fundamentally not clear whether this is applicable directly to BPMs. As far as we know, the second Wien effect has only been investigated as a non-equilibrium phenomenon over short times. In contrast, a BPM is generally operated in reverse bias at steady state. Another subtly occurs when considering electrochemical equilibrium or open-circuit voltage, *i.e.* conditions of no net current. Like a semiconductor *pn* junction, the BPM induces a junction electric field, even at equilibrium. If the second Wien effect plays a role here, does it mean that WD is accelerated at equilibrium? This would require that the ionization constant of water K_w is different in the region of large field from that in the field-free region. Answering these questions to cleanly elucidate WD mechanisms and catalysis in BPM junctions are important, but beyond the scope of this specific study.

However, we note that invoking the electric field alone as the source for the increased rate of WD does

not seem strictly necessary. A similar interpretation could invoke the *gradient in the electrochemical potential of the ionic charge carriers*, H^+ and OH^-. At electrochemical equilibrium an electric field – *i.e.* a gradient in electric potential – exists in the WD catalyst region, although the gradient in the electrochemical potential of all mobile species is, by definition, zero. This is because, conceptually, the free-energy contribution for the charged species from the electric potential is exactly balanced by the free-energy contribution from the concentration gradient across the BPM junction. If WD dissociation was accelerated by a gradient in electrochemical potential – *i.e.* total free energy per ionic species – rather than gradient in electric potential alone, then the WD rate would not be accelerated at equilibrium and K_w would be a constant across the BPM. A gradient in electrochemical potential could change the rate of WD because if the charged products, H^+ and OH^-, are generated some finite distance from each other. The larger the gradient, the larger the driving force for WD to spatially separated ionic products.

The above interpretation is similar to the general observation that (electro)chemical reaction rates increase exponentially with driving force. For example, in the simple Butler-Volmer model of charge transfer at an electrode surface, the rate of charge transfer increases exponentially with applied voltage. Some portion of applied potential serves to modulate the free energy of the products relative to the reactants and thus affects the transition state and activation energies. In Butler-Volmer theory, the overpotential driving the reaction is defined typically as $\eta = E - E_{eq}$, where E is the applied electrode potential and E_{eq} is the equilibrium electrode potential. This overpotential can be re-cast in terms of the interfacial electric potential drop $\Delta\phi$ across the double layer because the absolute number of electrons in a (metal) electrode changes little as the electrode potential is changed: $\eta = \Delta\phi - \Delta\phi_{eq}$. Like Butler-Volmer kinetics for electron transfer, we suspect that it is thus indeed not the magnitude of the interfacial electric field in the BPM that is important for accelerating WD, but in fact the local deviation from the equilibrium electric field at any given point within the junction. More work is needed to clarify these basic principles.

Comparison with previous work on electronically conducting materials. Chen *et al.*[8] presented the hypothesis that electronically conducting materials increase the electric field at the WD-catalyst/membrane interfaces to enhance WD. They used graphene and carbon nanotubes (CNT) as electronically conductive materials, and graphene oxide (GO) as an insulating WD catalyst. They tested two thickness (namely, “thin” and “thick”) for each catalyst. All of these three catalysts lower the voltage compared to the BPM without catalyst. From quantum-chemistry calculations of proton binding and release, they concluded that graphene and CNTs are not catalytic for WD. GO was found to be a better WD catalyst than either graphene or CNTs although it is an electronically insulating material. GO “thin” films provided better performance than “thick” films, yet for CNTs and graphene there was no apparent thickness dependence. Thus, they suggest that adding electronically conducting materials to a traditional catalyst could be strategy to improve performance, but do not directly demonstrate/prove the effect.

Here we tested key materials including electronic conductors, semiconductors, and insulators, each one with a quantitatively controlled loading/thickness range and, in some cases, different particle sizes and crystal structures. We show electronic conductors such as Pt and IrO_x achieve good performance at higher loadings compared with semiconducting TiO_2. Adding acetylene carbon black (ACB) to a thick, poorly performing TiO_2 layer substantially improved the performance. Since ACB itself is not catalytically active for WD, there is clearly synergistic effect between ACB and TiO_2. Presumably, TiO_2 provides the catalytic sites while ACB focuses the electric field at the interfaces. We also illustrate the weak role of ionic resistance for the oxide WD-catalyst interlayers and explain the U-shaped trend in voltage with catalyst loading.

Supplementary Table 1 Nanoparticles used as water dissociation (WD) catalysts.

Material	Supplier
Aeroxide® TiO_2-P25	Nippon Aerosil Co., Ltd.
TiO_2 anatase, 99.5% 5 nm	US Research Nanomaterials, Inc.
TiO_2 anatase, 99.5% 15 nm	US Research Nanomaterials, Inc.
TiO_2 anatase, high purity, 99.98% 30 nm	US Research Nanomaterials, Inc.
TiO_2 anatase, 99%, 100 nm	US Research Nanomaterials, Inc.
TiO_2 rutile, high purity, 99.9+%, 30 nm	US Research Nanomaterials, Inc.
SiO_2 99+%, 20-30 nm	US Research Nanomaterials, Inc.
Antimony tin oxide (ATO, SnO_2:Sb_2O_3 = 90:10, 30 nm, high purity, 99.95+%)	US Research Nanomaterials, Inc.
IrO_x	Pajarito Powder
Platinum black (high surface area)	Fuel Cell Store
Acetylene carbon black (99.99%, 50% compressed)	Strem Chemicals

Supplementary Table 2 BET surface areas and cumulative pore volumes based on the BJH method for TiO_2 nanoparticles.

Material	BET SSA (m^2 g^{-1})	Cumulative Pore Volume V_p (cm^3 g^{-1})
TiO_2-P25	92.6	0.703
TiO_2-Anatase 5 nm	285.9	0.596
TiO_2-Anatase 15 nm	77.7	0.368
TiO_2-Anatase 30 nm	53.3	0.297
TiO_2-Anatase 100 nm	14.3	0.038
TiO_2-Rutile 30 nm	23.0	0.054

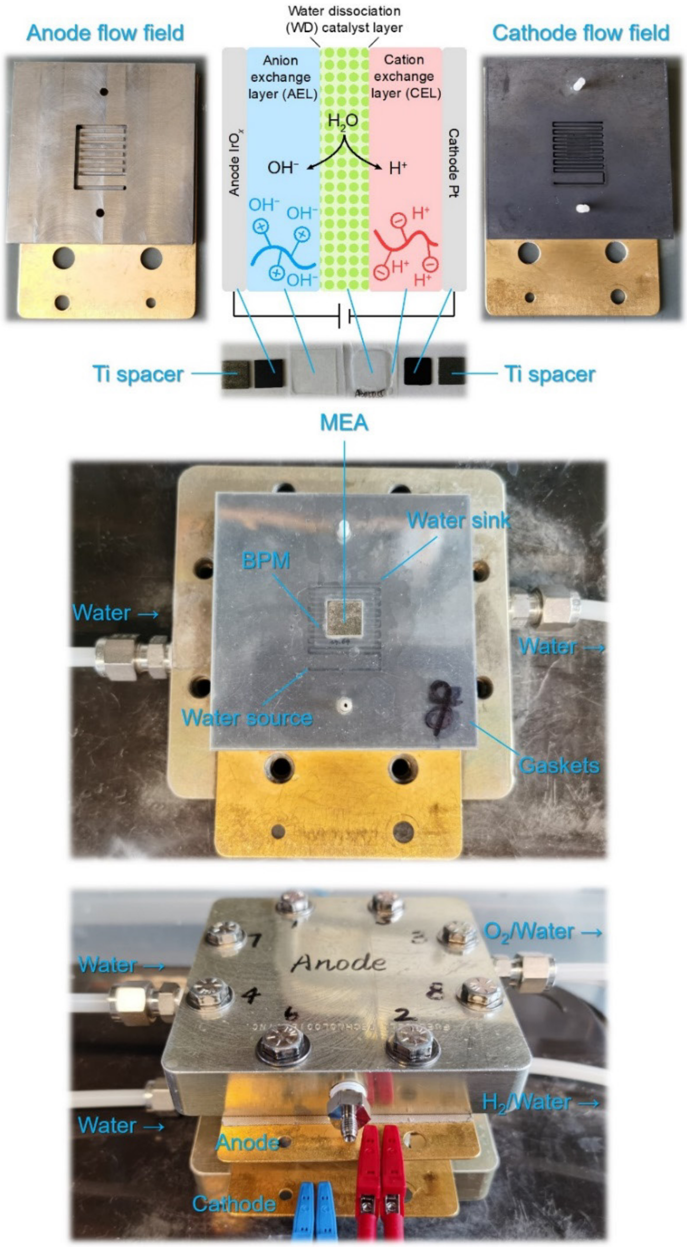

Supplementary Fig. 1 MEA electrolyzer setup. Pure water is fed into both anode and cathode. Water flows along the serpentines on the flow fields and through the porous Ti spacers and GDLs to reach the BPM. The flow fields also serve as points to attach the potentiostat power and voltage sense leads. The construction follows standard procedures from the membrane-electrolyzer and fuel-cell community, as described in the Methods section. The electrocatalysts, not to be confused with the *water dissociation* (WD) catalysts in the BPM, are mixed with ionomer and sprayed onto gas diffusion layers (GDLs) which provide electrical connection to catalyst. Impedance measurements are made also on this cell. As discussed in the text, there is a resistance due to the ionomer layer, as well as the membrane itself, and these result in a lumped high-frequency ohmic resistance (R_s in Fig. 5). The anode and cathode charge-transfer resistances and capacitances (R_{ct} and C_{ct}) are not easily separated. With our careful studies, however, we have rigorously identified and separated the impedance associated with the BPM junction (R_{wd}) where water is dissociated and transported to the CEL and AEL components of the BPM.

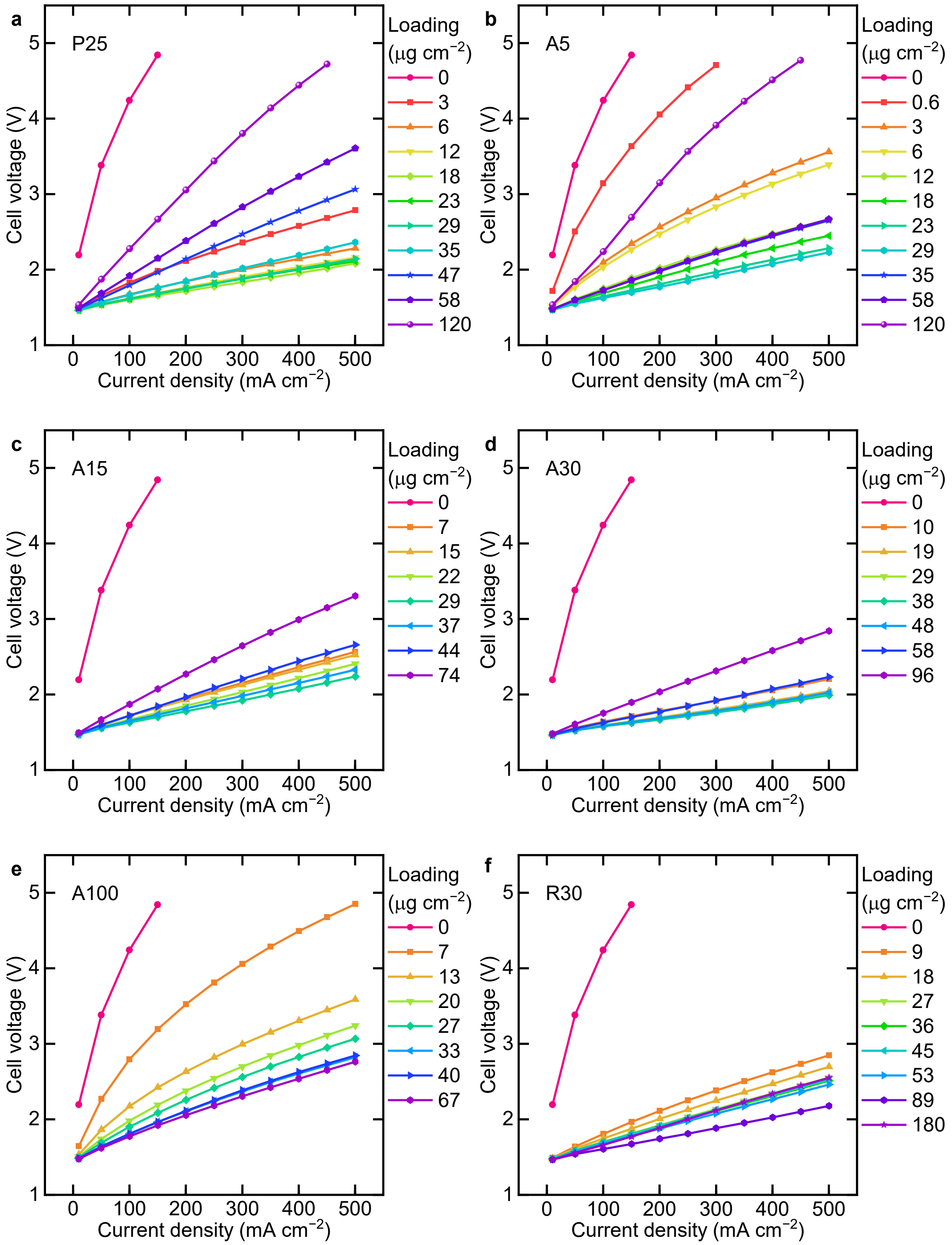

Supplementary Fig. 2 Performance of BPM electrolyzers with various TiO_2 as WD catalysts. Polarization curves of BPM electrolyzers with different loading of WD catalysts deposited by spray coating. **a**, TiO_2-P25. **b**, Anatase 5 nm. **c**, Anatase 15 nm. **d**, Anatase 30 nm. **e**, Anatase 100 nm. **f**, Rutile 30 nm.

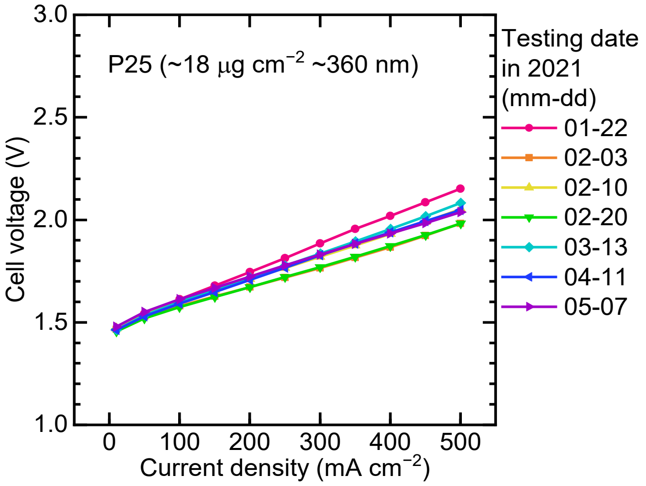

Supplementary Fig. 3 Reproducibility of electrolyzer performance of optimal TiO_2-P25 loading. TiO_2-P25 (~18 μg cm^{-2}) was deposited by spray coating. Tests were carried out at different dates with different batches of GDLs. The cell voltage at 500 mA cm^{-2} is 2.05 ± 0.06 V (standard error of 7 samples). The temperature is maintained 55 ± 2 °C. Benchmark AEM electrolyzers[9] with the same anode/cathode catalysts and Piperion AEM (and the same temperature) operate at ~1.85 V at 0.5 A cm^{-2}. This is only slightly worse than the BPM electrolyzer reported here, even though the BPM includes more membrane resistance from the use of a PEL and AEL in series, than the AEM alone, and must drive the WD reaction. This is because the BPM electrolyzers operates the cathode hydrogen evolution reaction in acid which is much faster than for the AEM electrolyzer which operates the cathode in locally alkaline conditions where the hydrogen evolution kinetics are much slower. This result therefore shows the substantial opportunity of BPM electrolyzer platforms to achieve good performance while also controlling the ion transfer and thus the local pH environment at each electrode.

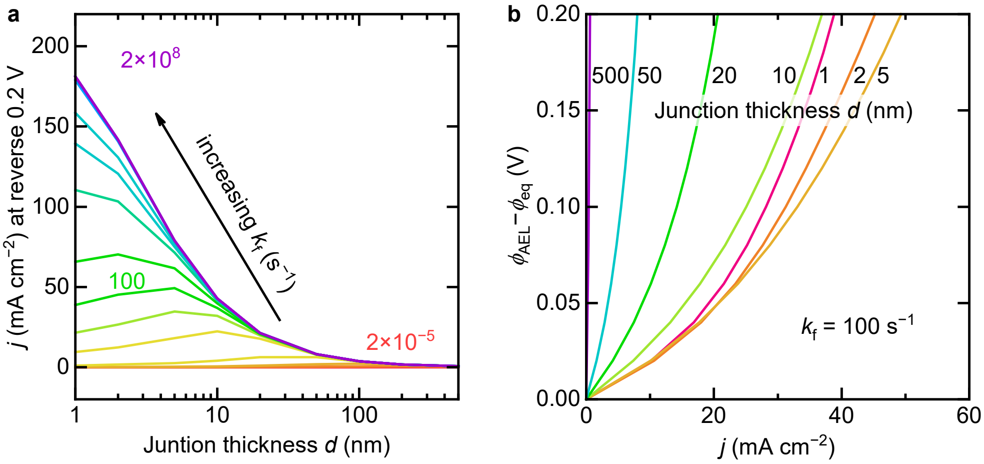

Supplementary Fig. 4 Steady-state numerical simulated results of BPMs with different junction thickness and WD rate constant using the reported diffusion coefficients for H^+ and OH^- along with the fixed ion concentration in the membranes estimated based on the manufacturer specifications. a, Current density at reverse bias 0.2 V as a function of junction thickness at different WD rate constant in the junction. **b**, Polarization curves in reverse bias of different junction thickness with WD rate constant k_f = 100 s^{-1}. See Methods for more information.

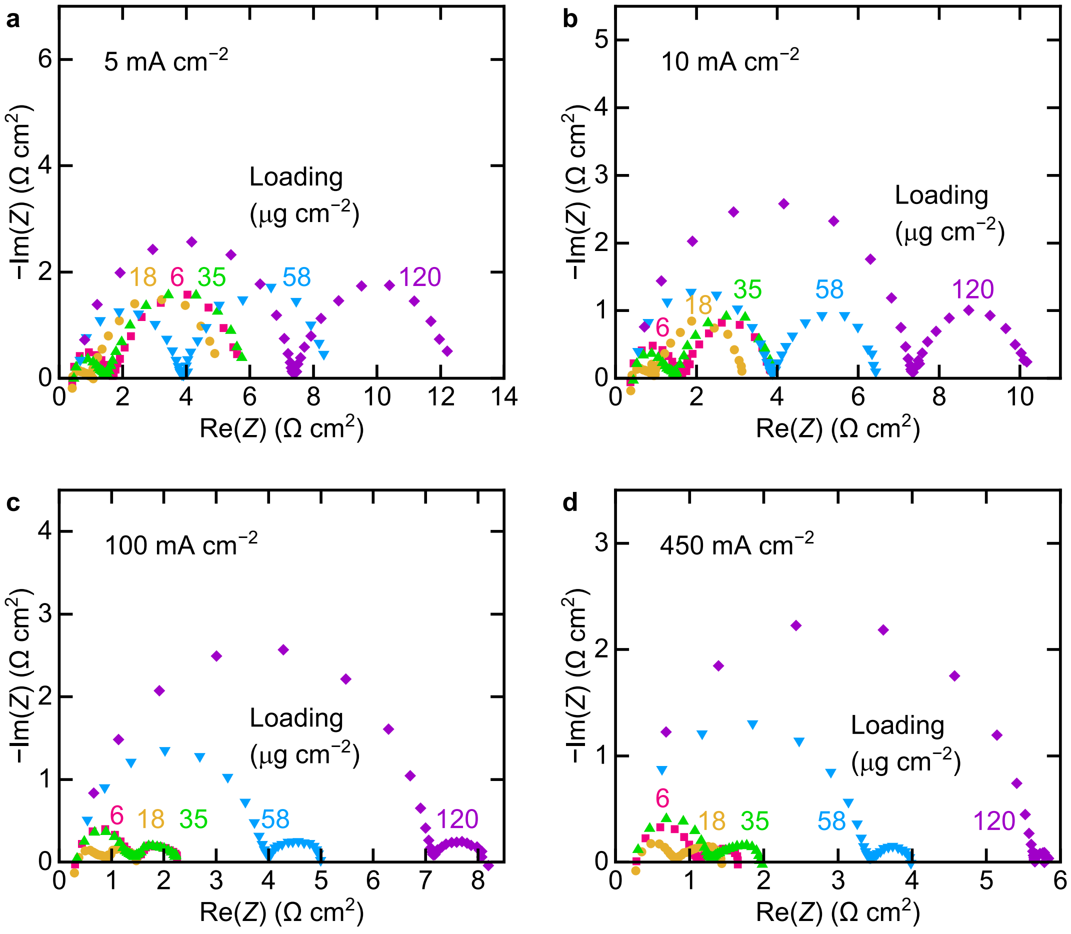

Supplementary Fig. 5 Nyquist plots of BPM electrolyzers at different current densities with different loadings of TiO_2-P25 WD catalysts deposited by spray coating. The high frequency semicircle is assigned to describe WD, while the low frequency one to describe charge transfer at the anode and cathode. **a**, 5 mA cm^{-2}. **b**, 10 mA cm^{-2}. **c**, 100 mA cm^{-2}. **d**, 450 mA cm^{-2}.

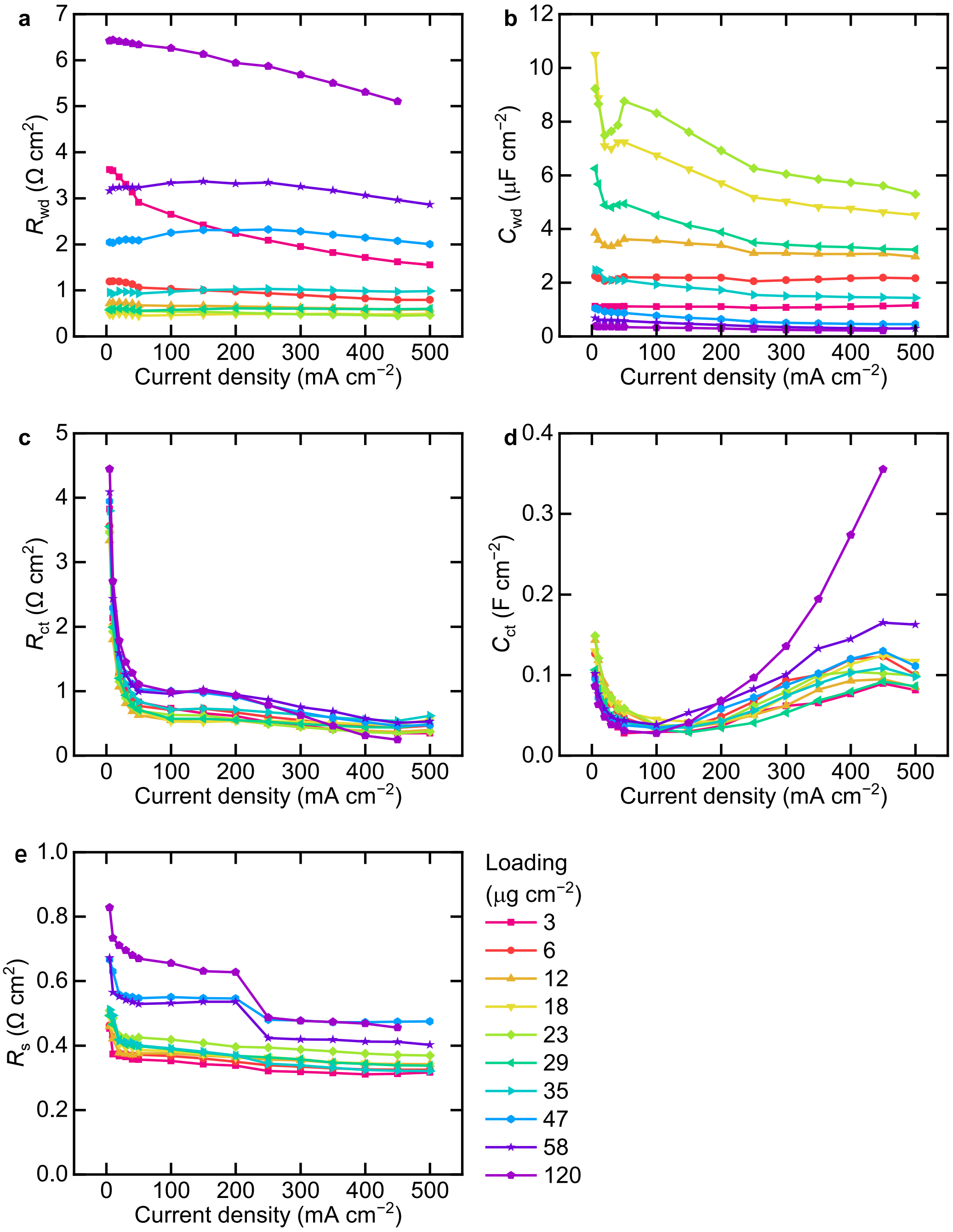

Supplementary Fig. 6 Impedance fitting results of the BPM electrolyzers with different loadings of TiO_2-P25 WD catalysts deposited by spray coating. a, WD resistance R_{wd}. **b**, WD capacitance C_{wd}. **c**, CT resistance R_{ct}. **d**, CT capacitance C_{ct}. **e**, Series resistance R_s.

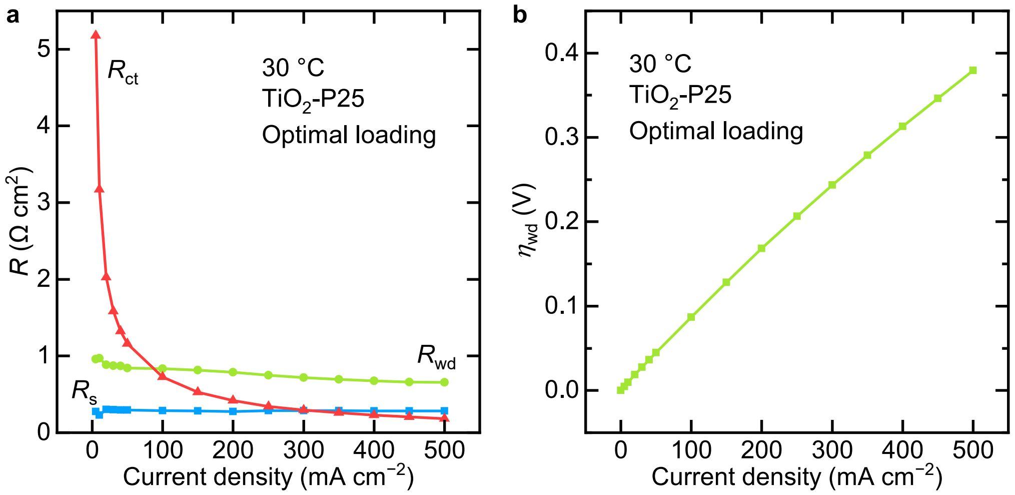

Supplementary Fig. 7 Impedance analysis and estimation of WD overpotential. **a**, Impedance fitting results of the BPM electrolyzers with optimal TiO_2-P25 loading (~18 μg cm^{-2} by spin coating) at 30 °C. **b**, Estimated WD overpotential $\eta_{\mathrm{wd}} = \int_0^j R_{\mathrm{wd}}(j)\mathrm{d}j$. The integral form of the total η_{wd} is necessary because the differential WD resistance R_{wd} depends on the voltage/current across the BPM junction. Much like related electrocatalytic processes, the differential resistance increases as driving force η_{wd} for the process increases.

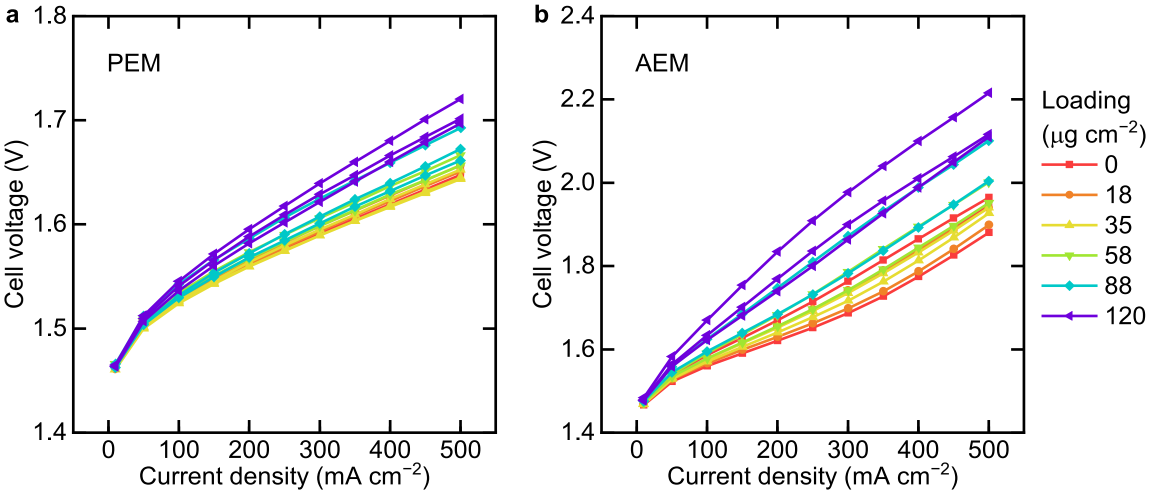

Supplementary Fig. 8 Performance of PEM and AEM electrolyzers. The TiO_2-P25 layers of different loadings were sandwiched between either two identical PEMs or AEMs. The region from 300 to 500 mA cm^{-2} is fitted to a line obtain the differential resistance. **a**, Polarization curves of PEM electrolyzers. **b**, Polarization curves of AEM electrolyzers.

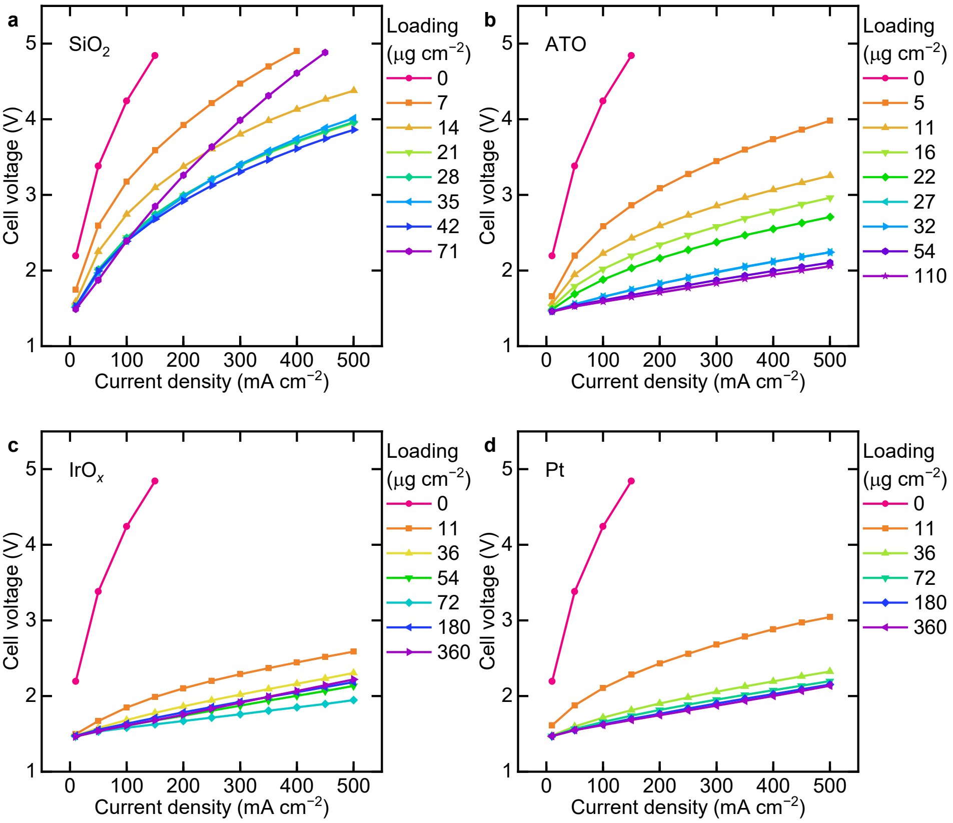

Supplementary Fig. 9 Performance of BPM electrolyzers with nanoparticles of different electronic conductivity as WD catalysts. Polarization curves of BPM electrolyzers with different loadings of WD catalysts deposited by spray coating. **a**, SiO_2. **b**, Antimony-doped tin oxide (ATO). **c**, IrO_x. **d**. Pt.

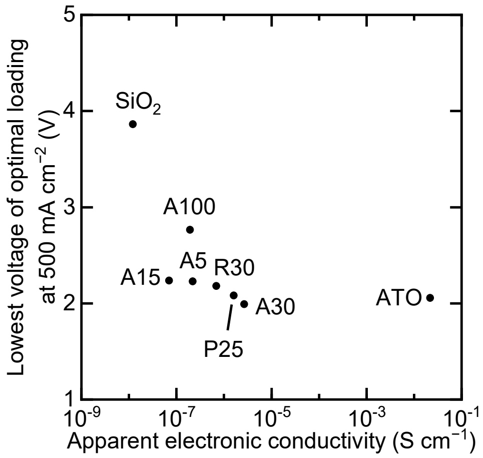

Supplementary Fig. 10 Relationship between BPM electrolyzer performance and the apparent electronic conductivities of WD catalysts. BPM electrolyzers performance is denoted by the lowest voltage with optimal WD catalyst mass loading at 500 mA cm^{-2}. The apparent electronic conductivities are measured using a simple two-probe setup (and thus includes any relevant contact resistances). For the TiO_2 samples, A = anatase, R = rutile, and the number denotes the size of the nanoparticles (nm) provided by the manufacturer. ATO = Sb:SnO_2.

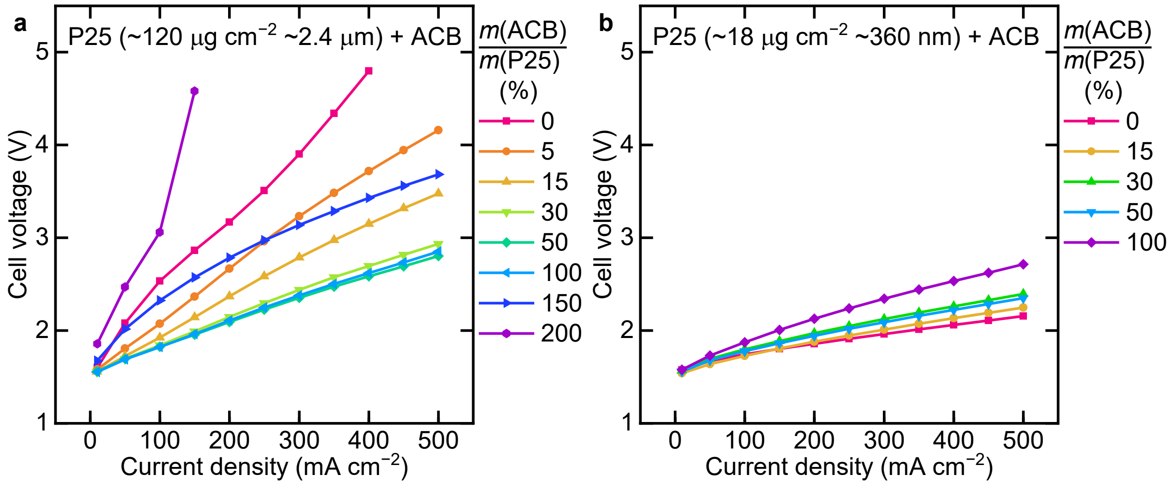

Supplementary Fig. 11 Performance of BPM electrolyzers with TiO_2-P25 mixed with acetylene carbon black (ACB) as WD catalysts. Polarization curves of BPM electrolyzers with different mass ratio of ACB and TiO_2-P25 deposited by spray coating. **a**, A thick layer of ~120 μg cm^{-2} (~2.4 μm) TiO_2-P25. **b**, A thin layer of TiO_2-P25 at optimal loading ~18 μg cm^{-2} (~360 nm).

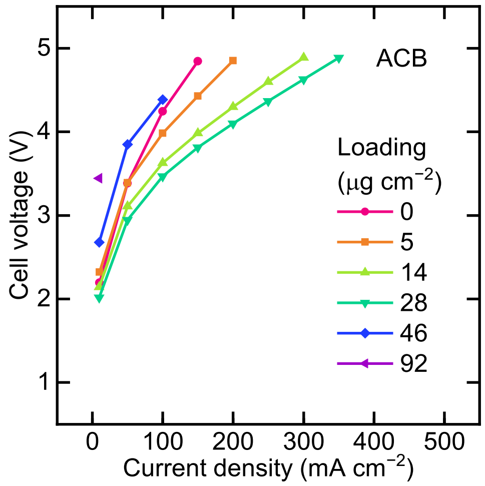

Supplementary Fig. 12 Performance of BPM electrolyzers with acetylene carbon black (ACB) as WD catalyst. Polarization curves of BPM electrolyzers with different loadings of WD catalysts deposited by spray coating.

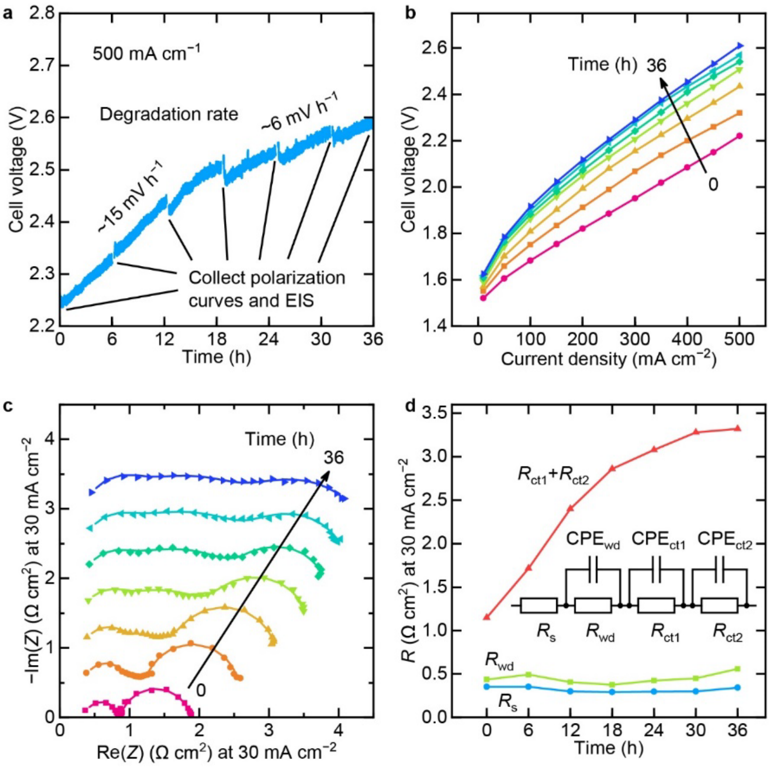

Supplementary Fig. 13 Stability of the BPM electrolyzer with the best loading (~18 μg cm^{-2}) of TiO_2-P25 at 500 mA cm^{-2} and 55 °C. a, Cell voltage at 500 mA cm^{-2} as a function of time. **b**, Polarization curves collected every 6 h. **c**, Nyquist plots collected at 30 mA cm^{-2} every 6 h. Dots are experimental data. Lines are fitted data. Curves are shifted 0.5 unit between datasets vertically for clarity. **d**. Fitted resistances change as a function of time. Inset is the equivalent circuit used to fit the EIS data. CPE = constant phase element. We checked the pH of the recirculating feed water before and after the 36 h stability test using both pH paper and a pH meter. The pH paper shows a pH of 6-7, the pH meter shows a pH of ~6.1, and there is no observable difference between the pH value before and after the stability test.

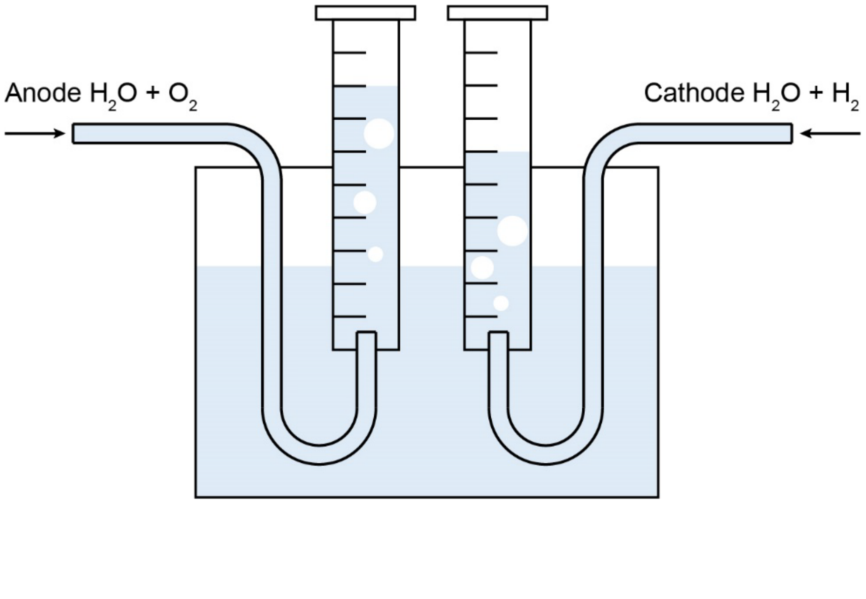

Supplementary Fig. 14 Schematic diagram of the apparatus to measure gas volume. The total volume of water is ~5.5 L. The volumes of the graduated cylinders are 50 mL for O_2 and 100 mL for H_2. The whole experiment is carried out in air, so the water already contains dissolved O_2 set by the partial pressure of O_2 in air (~0.21 atm). The gas bubbles are generated at the electrodes and carried by the fast water flow (~100 mL min^{-1} for anode and ~60 mL min^{-1} for cathode) to collection in the inverted graduated cylinders. Given the short transit time of between bubble detachment at the electrode and collection in the cylinder (~10 s), little O_2 is apparently lost due to dissolution in the recirculating water.

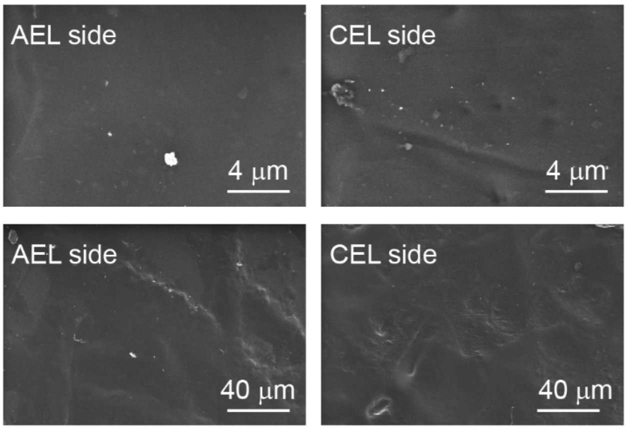

Supplementary Fig. 15 SEM images of a BPM after testing in the electrolyzer. The uneven morphology is due to the fiber texture of the GDLs and there is no evidence of cracks, pinholes, or other forms of mechanical failure of the ionomer membranes.

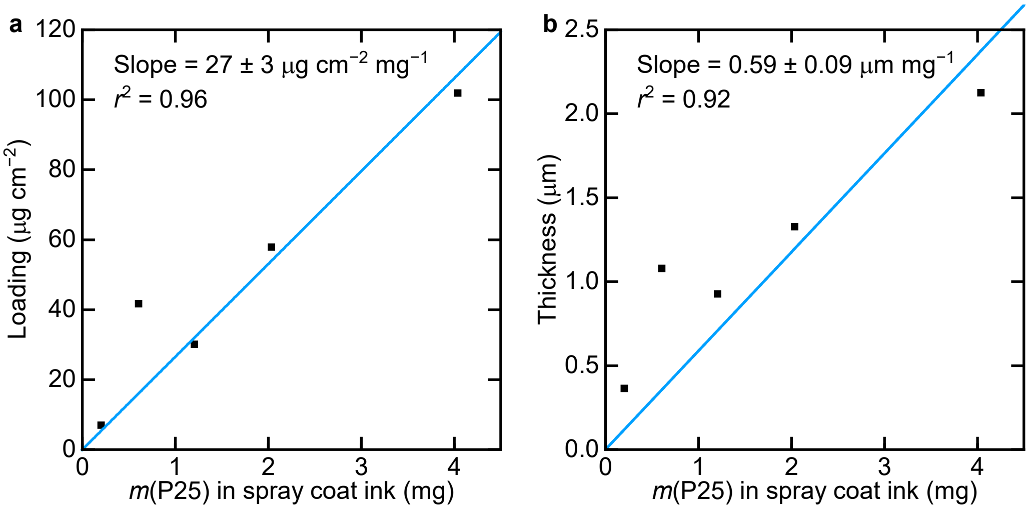

Supplementary Fig. 16 Quantifying loading and thickness of TiO_2-P25 in BPMs. The linear least squares fit to the calibration data provides the conversion factor used to predict loading and thickness given an amount of ink used and the mass of WD catalyst particles in the ink. This is important because a significant fraction of the ink is lost as overspray and thus does not end up in the BPM.

References

1 Giesbrecht, P. K. & Freund, M. S. Recent advances in bipolar membrane design and applications. *Chem. Mater.* **32**, 8060-8090, (2020).

2 Oener, S. Z., Foster, M. J. & Boettcher, S. W. Accelerating water dissociation in bipolar membranes and for electrocatalysis. *Science* **369**, 1099-1103, (2020).

3 Wien, M. Über eine Abweichung vom Ohmschen Gesetze bei Elektrolyten. *Ann. Phys.* **388**, 327-361, (1927).

4 Wien, M. Über den Spannungseffekt der Leitfähigkeit bei starken und schwachen Säuren. *Phys. Z.* **32**, 545-547, (1931).

5 Schiele, J. Über den Spannungseffekt der Leitfähigkeit bei starken und schwachen Säuren. *Ann. Phys.* **405**, 811-830, (1932).

6 Eckstrom, H. C. & Schmelzer, C. The Wien effect: Deviations of electrolytic solutions from Ohm's law under high field strengths. *Chem. Rev.* **24**, 367-414, (1939).

7 Onsager, L. Deviations from Ohm's law in weak electrolytes. *J. Chem. Phys.* **2**, 599-615, (1934).

8 Chen, Y., Martínez, R. J., Gervasio, D., Baygents, J. C. & Farrell, J. Water splitting promoted by electronically conducting interlayer material in bipolar membranes. *J. Appl. Electrochem.* **50**, 33-40, (2020).

9 Lindquist, G. A. *et al.* Performance and Durability of Pure-Water-Fed Anion Exchange Membrane Electrolyzers Using Baseline Materials and Operation. *ACS Appl. Mater. Interfaces* **13**, 51917-51924, (2021).
